# Cost-effectiveness of a precision hepatocellular carcinoma surveillance strategy in patients with cirrhosis

**DOI:** 10.1016/j.eclinm.2024.102755

**Published:** 2024-08-13

**Authors:** Szu-Yu Zoe Kao, Kinpritma Sangha, Naoto Fujiwara, Yujin Hoshida, Neehar D. Parikh, Amit G. Singal

**Affiliations:** a Siemens Medical Solutions USA Inc., Malvern, PA, USA; b Department of Internal Medicine, University of Texas Southwestern Medical Center, Dallas, TX, USA; c Department of Internal Medicine, University of Michigan, Ann Arbor, MI, USA

**Keywords:** Precision medicine, Liver cancer, Screening, Ultrasound, Biomarker, Abbreviated MRI

## Abstract

**Background:**

Hepatocellular carcinoma (HCC) surveillance is currently performed using a one-size-fits-all strategy with ultrasound plus AFP (US + AFP). There is increasing interest in risk-stratified and precision surveillance strategies incorporating individual risk and variance in surveillance test performance; however, the cost-effectiveness of these approaches has not been evaluated.

**Methods:**

We conducted a cost-effectiveness analysis to evaluate four surveillance strategies (no surveillance, universal US + AFP surveillance, risk-stratified surveillance, and precision surveillance) in a simulated cohort of 50-year-old patients with compensated cirrhosis. The most cost-effective strategy was that with the highest incremental cost-effectiveness ratio (ICER) and below the willingness-to-pay (WTP) threshold of $150,000/QALY gained. Model inputs were based on literature review, and costs were derived from the Medicare fee schedule.

**Findings:**

The precision surveillance strategy demonstrated variation in recommended surveillance test based on HCC risk category and patient factors. US + AFP, risk-stratified, and precision surveillance detected more HCC cases per 100,000 population than no surveillance, with a higher proportion of early-stage cases for precision surveillance (67.6%) than risk-stratified (63.8%), universal ultrasound (63.2%), and no surveillance (38.0%). Compared to no surveillance, precision surveillance was most cost-effective, with an ICER of $104,614/QALY gained, whereas US + AFP and risk-stratified surveillance were both dominated. Compared to US + AFP, risk-stratified surveillance was cost saving and dominated US + AFP, whereas precision surveillance was cost-effective, with an ICER of $98,103/QALY gained. Results were sensitive to survival with early-stage HCC, cost of early-stage HCC treatment, and surveillance utilization. Precision surveillance remained the most cost-effective when WTP thresholds exceeded $110,000/QALY gained.

**Interpretation:**

A precision surveillance strategy is the most cost-effective method for HCC surveillance. This approach could maximize surveillance benefits in high-risk patients, while minimizing surveillance harms in low-risk individuals.

**Funding:**

10.13039/100000054National Cancer Institute (U01 CA230694, R01 CA222900, R01 CA212008, and U24ca086368) and Cancer Prevention Research Institute of Texas (CPRIT) (RP200554).

**Research in context**

**Evidence before this study**

Professional society guidelines recommend hepatocellular carcinoma (HCC) surveillance using semi-annual abdominal ultrasound in all patients with cirrhosis, although there is variation in HCC risk and surveillance test performance by patient characteristics. The European Association for the Study of the Liver (EASL) released a policy statement advocating for precision surveillance with a goal of reducing deaths from HCC as well as associated costs, although there is a dearth of studies comparing this strategy to current practice.

**Added value of this study**

Precision surveillance would use varying surveillance tests in patients with cirrhosis based on HCC risk category and patient factors that can affect test accuracy. Precision HCC surveillance would detect a higher proportion of HCC at an early stage and be more cost-effective strategy than the current “one-size-fits-all” strategy using abdominal ultrasound as well as risk-stratified surveillance. Factors including cost of HCC treatment, survival with early-stage HCC, and surveillance utilization impact the cost-effectiveness of surveillance strategies.

**Implications of all the available evidence**

Implementation of precision surveillance may improve early detection of HCC and overall value compared to the current paradigm of abdominal ultrasound in all at-risk patients.

## Introduction

Hepatocellular carcinoma (HCC) is the fourth leading cause of cancer-related death globally and a leading cause of death in patients with compensated cirrhosis.^1^ HCC is one of the few cancers with a 5-year survival that remains below 20%, although prognosis markedly differs by tumor stage. Patients with early-stage HCC are amenable to curative treatments, yielding a median survival exceeding 10 years, whereas those with advanced tumor burden have a median survival of 1–3 years.^2^ Given the association with improved clinical outcomes, the European Association for the Study of the Liver (EASL) and American Association for Study of Liver Diseases (AASLD) recommend semi-annual HCC surveillance in at-risk populations.^3^^,^^4^ Surveillance is associated with improved early HCC detection and patient survival as demonstrated by a large, randomized control trial among patients with hepatitis B virus (HBV) and several cohort studies in patients with cirrhosis.^5^^,^^6^

Surveillance has traditionally been completed using abdominal ultrasound, which has several advantages including wide availability, low cost, non-invasiveness, and overall favorable safety profile. However, ultrasound has suboptimal sensitivity for early HCC detection, particularly in patients with metabolic dysfunction associated steatotic liver disease (MASLD) or alcohol-associated liver disease (ALD).^7^ Obesity and non-viral etiologies of cirrhosis are both associated with increased odds of suboptimal visualization, which in turn is associated with lower sensitivity for early-stage HCC detection.^8^^,^^9^

Consequently, there is growing interest in blood- and alternate imaging-based modalities to address these limitations.^10^ For example, dynamic contrast enhanced MRI was demonstrated to have significantly higher sensitivity and specificity compared to ultrasound in a cohort of patients with HBV-related cirrhosis.^11^ Although concerns about cost and imaging capacity may limit widespread adoption of complete MRI for HCC surveillance, abbreviated MRI (AMRI) protocols have been proposed to decrease in-scanner time, with early data suggesting preserved test performance.^12^^,^^13^ In parallel, there is increasing data for validation of emerging blood-based biomarker panels, with promising test performance in biomarker phase 2 case–control studies.^14^^,^^15^ The best validated biomarker panel to date is GALAD, which combines gender, age, and 3 biomarkers (AFP, AFP-L3, and DCP). GALAD demonstrated sensitivities of 60–80% for early-stage HCC detection in a multi-national case–control study and was shown to have a sensitivity and specificity of 65% and 82%, respectively, in the phase 3 Early Detection Research Network (EDRN)-funded HCC Early Detection Strategy (HEDS) Study.^16^

Most studies examining surveillance modalities, including cost-effectiveness analyses, have examined them in a one-size-fits-all manner for at-risk patients.^17^ However, HCC risk varies among patients with chronic HBV infection or cirrhosis, with several widely recognized risk factors such as older age, male sex, obesity, active viremic disease, and increased liver disease severity.^18^ Further, surveillance test performance can differ across patient subgroups. For example, AMRI may have lower sensitivity for early-stage HCC detection in patients with decompensated cirrhosis and GALAD may have lower sensitivity in women than men.^13^^,^^15^ Accordingly, there has been increased interest in precision surveillance, whereby the optimal surveillance test is tailored to an individual's HCC risk and anticipated test performance. Indeed, EASL recently released a policy statement advocating for precision surveillance with a goal of reducing deaths from HCC as well as associated costs.^19^ The aim of our study was to compare the cost and effectiveness between one-size-fits-all, risk-stratified, and precision surveillance in a cohort of patients with cirrhosis.

## Methods

### Overview

We developed a microsimulation model to evaluate the cost-effectiveness of HCC surveillance strategies in a cohort of 50-year-old with compensated cirrhosis over a lifetime with monthly cycles. All patients were without prior history of HCC at baseline. The model incorporated heterogeneity in the population by considering patient risk groups, which are stratified by the annual risk of incident HCC (low-, intermediate-, and high-risk), and HCC risk factors, which can influence surveillance sensitivity and specificity. A cost-effectiveness analysis (CEA) was conducted to compare four semiannual HCC surveillance strategies from the US healthcare perspective: (1) *no surveillance*; (2) *universal ultrasound + AFP surveillance* for all patients; (3) *risk-stratified surveillance* in which the surveillance strategy varied by patient risk group; and (4) *precision surveillance* in which the surveillance strategy varied by risk group and patient factors that can affect test performance. Model simulations were performed in TreeAge Pro (V.2023, TreeAge Software, Williamstown, Massachusetts, USA). Post-simulation analyses including CEA and sensitivity analyses were conducted using dampack package in R 4.2.2.^20^ Institutional review board was not required because no patient data were involved in analyses.

### Natural disease progression

The state–transition diagram is presented in Figure S1, and input parameters are provided in Table 1. Patients with compensated cirrhosis could develop decompensated cirrhosis (5% annual risk), HCC, or death within each cycle.^17^^,^^21^^,^^22^ The annual mortality rate for patients with compensated cirrhosis without HCC was accounted for using the background mortality rate determined by the US life table and an annual excess mortality rate due to compensated cirrhosis at 4%.^17^^,^^21^^,^^22^^,^^42^ Annual risk of incident HCC was stratified into low (0.5% annual incidence), intermediate (1.5% annual incidence), and high (5.0% annual incidence) risk groups among patients with compensated cirrhosis; annual HCC risk estimates were based on published clinical and biomarker-based models.^43^^,^^44^ Patients with decompensated cirrhosis had an annual risk of developing HCC of 4% across risk groups.^17^^,^^22^

**Table 1:** Values, ranges, and sources of input parameters.

Input parameters	Base case	Range	Distribution	Sources
**Natural disease progression (without intervention)**				
Annual probability of disease progression from compensated to decompensated cirrhosis	5.0%	3%–8%	Beta (11.635, 216.508)	^17^^,^^21^^,^^22^
Annual probability of developing HCC from compensated cirrhosis				^23^
Low-risk	0.5%			
Intermediate-risk	1.5%			
High-risk	5.0%			
Annual probability of developing HCC from decompensated cirrhosis	4%	2%–6%	Beta (17.092, 399.791)	^21^^,^^22^
Annual rate of disease progression from BCLC A to BCLC B	90%	85%–95%	Beta (148.405, 16.489)	24, 25, 26
Annual rate of disease progression from BCLC B to BCLC C	80%	70%–90%	Beta (56.624, 14.156)	24, 25, 26
Annual rate of disease progression from an earlier BCLC stage to BCLC D				
Compensated BCLC A → BCLC D	0.5%	0.0%–1.5%	Beta (0.007, 1.343)	24, 25, 26
Compensated BCLC B → BCLC D	1.5%	0.5%–2.0%	Beta (38.798, 2547.745)	24, 25, 26
Compensated BCLC C → BCLC D	2.5%	1.0%–4.0%	Beta (13.035, 508.356)	24, 25, 26
Decompensated BCLC A → BCLC D	2.0%	1.0%–4.0%	Beta (5.280, 258.726)	24, 25, 26
Decompensated BCLC B → BCLC D	3.0%	2.0%–5.0%	Beta (10.726, 346.821)	24, 25, 26
Decompensated BCLC C → BCLC D	4.0%	3.0%–6.0%	Beta (17.891, 429.389)	^22^^,^^24^, ^25^, ^26^, ^27^
**Surveillance- or diagnosis-related parameters**				
Diagnostic MRI				
Sensitivity				
BCLC stage A	74%	50%–89%	Beta (11.130, 3.911)	^28^
BCLC stages B-D	95%	92%–99%	Beta (240.023, 12.633)	^29^
Specificity	94%	85%–98%	Beta (38.066, 2.430)	^29^
Probability of false positive results that led to diagnostic MRIs				
Ultrasound plus AFP or biomarker	8%	5%–11%	Beta (28.995, 333.441)	^30^^,^^31^
AMRI	6%	3%–10%	Beta (10.291, 161.231)	^32^
Number of diagnostic MRIs completed for true positive cases	1.5	1–3	Poisson (1.5)	^30^^,^^31^
Number of diagnostic MRIs completed for false positive cases				
Ultrasound plus AFP or biomarker	1.2	1.0–1.5	Poisson (1.2)	^30^^,^^31^
AMRI	2	1.5–3	Poisson (2)	^12^
Biopsy				
Probability of diagnostic MRIs leading to biopsy due to indeterminant results				
BCLC stage A	3%	1%–5%	Beta (10.720, 346.607)	^30^^,^^31^
BCLC stages B-D	10%	5%–20%	Beta (4.647, 41.821)	^30^^,^^31^
Biopsy bleeding or biliary injury	0.60%	0.3%–0.9%	Beta (18.535, 3070.600)	^30^
Death from biopsy	0.08%	0.04%–0.10%	Beta (63.948, 79871.052)	^30^
False negative biopsy results				
BCLC stage A	30%	22%–36%	Beta (70.509, 164.521)	^30^^,^^33^
BCLC stages B-D	5%	3%–15%	Beta (1.502, 28.543)	^33^
**Survival estimates**				
Annual excess mortality for compensated cirrhosis	4%	1.8%–8%	Beta (5.121, 122.906)	^21^^,^^22^
Median survival by diagnosis status (years)				
Decompensated cirrhosis	2.5	0.5–5.0	Gamma (5.430, 2.172)	^22^
Compensated BCLC stage A				
Undiagnosed^a^	2.9	1.0–4.0	Gamma (4.000, 2.274) + 1	^2^^,^^24^
Diagnosed	8.0	1.0–19.0	Gamma (4.000, 0.500)	^2^
Compensated BCLC stage B				
Undiagnosed^a^	1.5	0.08–3.0	Gamma (44.444, 30.296) + 1	24, 25, 26
Diagnosed	3.7	0.08–8.5	Gamma (4.000, 1.081)	^2^
Compensated BCLC stage C				
Undiagnosed^a^	0.75	0.5–1.2	Gamma (11.111, 11.111) + 1	24, 25, 26
Diagnosed	1.5	1.0–3.0	Gamma (5.430, 3.620)	^2^
Decompensated BCLC stage A				
Undiagnosed^b^	2.5			Calculated
Diagnosed^b^	2.5			Calculated
Decompensated BCLC stage B				
Undiagnosed^b^	1.5			Calculated
Diagnosed^b^	2.5			Calculated
Decompensated BCLC stage C				
Undiagnosed^b^	0.8			Calculated
Diagnosed^b^	1.5			Calculated
Diagnosed/undiagnosed BCLC stage D	0.5	0.25–0.75	Gamma (18.809, 37.617)	^2^
**Cost (2021 US Dollars)**				
Surveillance/diagnostic costs (one-time cost)				
Abdominal US	$158	$148–$216	Gamma (33.548, 0.212)	Medicare fee schedule
AFP	$21	$18–$23	Gamma (327.500, 15.800)	Medicare fee schedule
Biomarker (GALAD)	$200	$116–$600	LogNormal (5.070, 0.676)	Medicare fee schedule
MRI Abdomen with and without contrast	$554	$490–$720	Gamma (48.908, 0.088)	Medicare fee schedule
AMRI	$453	$310–$605	Gamma (39.100, 0.090)	Medicare fee schedule
Liver biopsy	$1149	$939–$1358	Gamma (124.490, 0.110)	Medicare fee schedule
Liver biopsy complications	$6048	$1537–$39,153	LogNormal (7.372, 1.634)	^34^
Average post-diagnosis annual medical cost by disease stage				
BCLC staging				
BCLC A	$50,001	$26,777–$92,737	Gamma (7.160, 0.000)	^35^^,^^36^
BCLC B	$93,221	$42,254–$222,397	LogNormal (11.313, 0.510)	^35^^,^^37^
BCLC C	$84,342	$38,230–$192,070	LogNormal (11.228, 0.478)	^35^^,^^37^
Compensated cirrhosis	$3832	$1233–$11,308	LogNormal (8.030, 0.665)	^35^^,^^37^
Decompensated cirrhosis	$22,000	$4316–$39,578	LogNormal (9.801, 0.665)	^38^
Palliative care (daily)	$214	$168–$826	LogNormal (4.971, 0.890)	Medicare fee schedule
**Utilities**				
Utility increment for undiagnosed disease state	0.05	0.00–0.10	PERT (min = 0.00, mode = 0.05, max = 0.10, shape = 4)	Assumption
Compensated cirrhosis	0.85	0.68–0.98	Beta (19.141, 3.378)	^39^^,^^40^
Compensated BCLC stage A				
Diagnosed	0.72	0.62–0.82	Beta (60.551, 23.547)	^39^^,^^40^
Undiagnosed	0.77	0.67–0.87	Diagnosed + utility increment	^39^^,^^40^^;^ calculated
Compensated BCLC stage B				
Diagnosed	0.69	0.62–0.78	Beta (121.648, 54.653)	^39^^,^^40^
Undiagnosed	0.74	0.67–0.83	Diagnosed + utility increment	^39^^,^^40^^;^ calculated
Compensated BCLC stage C				
Diagnosed	0.65	0.52–0.78	Beta (35.484, 19.107)	^39^^,^^40^
Undiagnosed	0.7	0.57–0.83	Diagnosed + utility increment	^39^^,^^40^^;^ calculated
BCLC stage D				
Diagnosed with best supportive care	0.4	0.37–0.42	Beta (927.038, 1390.557)	^41^
Undiagnosed	0.62	0.51–0.73	Diagnosed + utility increment	^39^^,^^40^^;^ assumption
Any HCC stages with cirrhosis decompensation				
Diagnosed	0.57	0.46–0.68	Beta (44.902, 33.874)	^39^^,^^40^
Undiagnosed	0.62	0.51–0.73	Diagnosed + utility increment	^39^^,^^40^^;^ calculated
Decompensated cirrhosis	0.78	0.53–0.93	Beta (10.222, 2.883)	^39^^,^^40^
**Other**				
Background mortality	Age and sex–specific			2019 US life table

AFP, alpha fetoprotein; BCLC, Barcelona Clinic Liver Cancer; GALAD, gender, age, AFP-L3, AFP, DCP; HCC, hepatocellular carcinoma; MRI, magnetic resonance imaging; PERT, program evaluation and review technique.

a The distribution used in the probabilistic sensitivity analysis (PSA) was set to be greater than 1 and was not directly used in the PSA. In the PSA, the sample of the survival estimate for undiagnosed disease state was calculated as the survival estimate for the corresponding diagnosed disease state divided by the distribution presented in the table to guarantee that the survival of a diagnosed disease state was longer than that of the corresponding undiagnosed disease state.

b Minimum of the survival estimates associated with relevant disease states and diagnosis status.

HCC could be detected incidentally, symptomatically, or by surveillance. The probability of incidental detection was estimated by calibration for early stage HCC (Barcelona Clinical Liver Cancer [BCLC] 0-A) based on published estimates of incidental vs. surveillance detected HCC.^23^^,^^45^ Details regarding the probabilities of incidental detection are included in Supplementary Methods, Table S1. Survival for each tumor stage and for patients with decompensated cirrhosis was determined from the literature and described in Supplementary Methods, Table S2.^46^, ^47^, ^48^, ^49^ Within each cycle, HCC could progress to more advanced tumor stages (e.g, BCLC 0-A to B or BCLC B to C).^50^ Additionally, patients could develop decompensated cirrhosis (Child C) or poor performance status (ECOG >2), and thereby progress directly to BCLC D. The annual rate of HCC stage migration varied with initial BCLC stage and degree of liver dysfunction.^51^

All annual probabilities and rates were converted to monthly transition probabilities following the assumption of declining exponential approximation: $probability=1-\exp\left(-\frac{rate}{12}\right)$.^52^ Additionally, estimates of median survival time was transformed to annual rates as ln (2)/time.

### Surveillance strategies

As described above, we compared four surveillance strategies: no surveillance, universal ultrasound plus AFP, risk-stratified surveillance, and precision surveillance. *Universal ultrasound plus AFP surveillance* mirrored current recommendations, by which all patients with cirrhosis underwent semi-annual ultrasound plus AFP regardless of individual risk and patient profile.^3^^,^^4^ *Risk-stratified surveillance* comprised no surveillance for low-risk patients, ultrasound plus AFP for intermediate-risk patients, and AMRI for high-risk patients. The choice of surveillance modality for each risk group was based on prior cost-effectiveness models.^11^^,^^53^ *Precision surveillance* allowed surveillance strategies to vary with both risk group and four patient factors that have been shown to impact surveillance test performance: patient sex (male vs. female), etiology of liver disease (viral vs. non-viral), Child-Pugh class (A vs. B/C), and body mass index (obese vs. non-obese).^9^^,^^13^^,^^14^ The combination of the three risk groups and four patient factors yielded 48 patient types. The overall *Precision surveillance* strategy consisted of the most cost-effective testing strategy for each of the 48 patient types, with four potential surveillance strategies considered (no surveillance, semi-annual US + AFP, semi-annual GALAD, and semi-annual AMRI) (see Supplemental Methods for details). The estimates of population weights for each risk group and patient factor are summarized in Table S3. In compensated cirrhosis patients, 63% were males, 51% were obese, 64% were classified as Child-Pugh A, and 39% had viral cirrhosis.^54^^,^^55^ The distribution of patient risk groups was 23% low-risk, 56% intermediate-risk, and 21% high-risk.^54^^,^^56^

Surveillance results were modeled as a function of the test performance (sensitivity, specificity), which varied by surveillance modality and patient characteristics. In the literature, test sensitivity and specificity were typically reported as a marginal estimate for each risk factor (e.g., sensitivity of US + AFP for males), instead of an estimate accounting for all four risk factors simultaneously (e.g., sensitivity of US + AFP for males with viral cirrhosis, Child-Pugh A, and obesity). To account for all four risk factors, we used the minimum among all four marginal estimates associated with the specific patient type. Estimates of test sensitivity and specificity, overall and stratified by patient type, are detailed in Supplementary Methods, Table S4, and Figure S2.

The diagnostic recall pathway is outlined in Figure S3 and input parameters are provided in Table 1. Patients with true negative surveillance results returned to their health states and could remain HCC-free, whereas those with false negative results progressed to more advanced tumor stage in the absence of treatment. Patients with positive or indeterminate surveillance results underwent diagnostic MRI, although some patients required repeated diagnostic MRI to establish a diagnosis (e.g., indeterminate liver nodule on first diagnostic imaging).^30^^,^^31^ False positive cases were resolved by repeated diagnostic MRIs. Biopsy was performed in some patients with indeterminant diagnostic MRI results and could result in true positive or false negative results.^30^^,^^31^

### Costs, utilities, and outcomes

Costs and utilities were calculated from the US healthcare perspective (Table 1). Costs were derived from the CMS payment rates or published studies, including both short-term (e.g., surveillance, diagnostic tests, biopsy, complications) and long-term costs.^17^^,^^34^^,^^57^ All costs were inflation adjusted to 2021 US dollars. Utilities for diagnosed disease states were parameterized from the literature.^39^^,^^41^ Utility for an undiagnosed disease state was estimated to be higher than the corresponding disease state by a utility increment of 0.05. The primary outcomes in this study were lifetime costs and quality-adjusted life years (QALYs) discounted at an annual rate of 3% for each surveillance strategy.^58^ Secondary outcomes included lifetime prevalence of HCC, number of surveillance tests provided, number of HCC cases detected, distribution of stage at HCC diagnosis, and number of surveillance-detected cases.

### Simulation and cost-effectiveness analysis

We simulated a cohort of 500,000 cirrhosis patients by sampling individuals from the distribution of patient types according to the population weights. For each simulated individual, the input parameters such as the risk of incident HCC and test performance for each surveillance strategy were extracted and determined by the individual's risk group and factors.

In CEA, all strategies were ranked in ascending order of cost, from the least to most costly. The incremental cost-effectiveness ratio (ICER) of a strategy was calculated as the ratio of incremental costs to incremental QALYs between the strategy and the previous less costly strategy. Strongly and weakly dominated strategies were identified and excluded from the analysis. A strongly dominated strategy is defined as a strategy with fewer QALYs at a higher cost than the previous less costly strategy; a weakly dominated strategy is when the ICER of the strategy is higher than that of the next expensive strategy. We compared the ICER of each surveillance strategy with a willingness-to-pay (WTP) threshold of $150,000/QALY gained, which was used to account for proposed increase in cost-effectiveness thresholds in the U.S.^59^^,^^60^ Three comparisons were conducted, including (1) comparison among all four strategies; (2) head-to-head comparisons between each surveillance strategy vs. no surveillance; (3) comparison among the three active surveillance strategies with universal US + AFP surveillance as the reference, excluding no surveillance.

### Sensitivity analyses

Deterministic one-way sensitivity analyses were performed to assess how CEA results changed over parameter values within the ranges determined from the literature. In one-way sensitivity analyses, strategies were compared based on the net monetary benefit (NMB) of each strategy, which represents net health benefits in monetary unit calculated as WTP x QALYs − costc. A strategy with a higher NMB indicates that it is more cost-effective than a strategy with a lower NMB. A three-way sensitivity analysis was conducted to evaluate how cost-effectiveness changed with varying adherence for each modality. A probabilistic sensitivity analysis (PSA) was also conducted to evaluate how likely a strategy is cost-effective under parameter uncertainty. Uncertainty of a parameter was captured using statistical distribution by fitting its mean to the base case value and its 95% intervals to the reported parameter range. PSA was conducted using 1000 sample sets generated from the parameter distributions. For each sample set, a cohort of 100,000 patients was simulated to obtain stable estimates of costs and QALYs. A cost-effective acceptability curve (CEAC) including all four strategies was produced to calculate the percent of PSA samples that a surveillance strategy was cost-effective at various WTP thresholds ranging from $1000 to $500,000/QALY gained. We also produced a CEAC showing a head-to-head comparison between no surveillance and each surveillance strategy.

### Role of the funding source

The funders of the study had no role in study design, data analysis, data interpretation, or writing of the manuscript. The corresponding author had full access to all study data and has final responsibility for the decision to submit for publication.

## Results

### Precision surveillance strategy

The cost-effective surveillance strategies by patient type for precision surveillance are presented in Figure S4. In brief, no surveillance was preferred for most low-risk female patients except for non-obese individuals with viral-related, Child-Pugh A cirrhosis, whereas GALAD was preferred for male patients. For intermediate-risk patients, US + AFP and AMRI were optimal for female patients depending on the patient type (e.g., AMRI preferred for obese patients with Child-Pugh A cirrhosis), and GALAD was optimal for male patients. For high-risk patients, AMRI was optimal for most female patients except for those with viral cirrhosis, Child-Pugh B/C, and non-obese, for whom US + AFP was recommended. In comparison, AMRI was optimal for high-risk male patients with Child-Pugh A cirrhosis and GALAD was preferred for high-risk male patients with Child-Pugh B or C cirrhosis.

### Simulation outcomes

The simulation outcomes by surveillance strategy are reported in Table 2. All active surveillance strategies detected more HCC cases/100,000 population (universal US + AFP: 17,387 cases; risk-stratified: 16,859 cases, precision: 17,403 cases) than the no surveillance strategy (11,650 cases/100,000 population). HCC detection was proportional to the number of tests offered, with universal US + AFP detecting the highest proportion of HCC cases (81.5%) by offering the most tests (1,899,075 tests/100,000 population) and risk-stratified surveillance detecting the fewest cases (76.2%) by providing the least number of tests (1,404,947 tests/100,000 population). The proportion of HCC cases diagnosed at an early-stage (BCLC 0-A) was 38.0% for no surveillance, which increased to 63.2% for universal US + AFP, 63.8% for risk-stratified surveillance, and 67.6% for precision surveillance. Details regarding tumor stage and mode of HCC detection for each of the active surveillance strategies are illustrated in Figure S5.

**Table 2:** The mean and 95% credible intervals (brackets) of simulated outcomes.

Outcome	Estimate [95% interval]			
	No surveillance	Universal US + AFP surveillance	Risk-stratified surveillance	Precision surveillance
Lifetime prevalence of HCC	20.8% [20.7%–20.9%]	20.8% [20.6%–20.9%]	20.8% [20.6%–20.9%]	20.7% [20.6%–20.9%]
Total number of surveillance tests per 100,000 population	–	1,899,075 [1,894,575–1,903,574]	1,404,947 [1,400,556–1,409,337]	1,697,193 [1,692,703–1,701,682]
Number of HCC cases detected per 100,000 population	11,650 [11,480–11,820]	17,387 [17,141–17,633]	16,859 [16,607–17,110]	17,403 [17,158–17,649]
Mode of detection, %				
Incidental detection	100.0%	18.5%	23.8%	18.7%
Surveillance detection	0.0%	81.5%	76.2%	81.3%
Stage at diagnosis, N (%)				
Stage A	4434 (38.0%)	10,972 (63.2%)	10,752 (63.8%)	11,754 (67.6%)
Stage B	4355 (37.4%)	5573 (32.0%)	5073 (30.0%)	4805 (27.6%)
Stage C	2699 (23.2%)	772 (4.4%)	953 (5.6%)	773 (4.4%)
Stage D	161 (1.4%)	70 (0.4%)	80 (0.4%)	71 (0.4%)
Risk group, N (%)				
Low-risk				
Early stage (stage A)	508 (37.2%)	1319 (63.6%)	508 (37.2%)	1046 (58.6%)
Late stages (stages B-D)	856 (62.8%)	756 (36.4%)	856 (62.8%)	739 (41.4%)
Intermediate-risk				
Early stage (stage A)	2203 (38.2%)	5428 (63.2%)	5428 (63.2%)	5885 (67.5%)
Late stages (stages B-D)	3571 (61.8%)	3157 (36.8%)	3157 (36.8%)	2835 (32.5%)
High-risk				
Early stage (stage A)	1723 (38.2%)	4225 (62.8%)	4816 (69.7%)	4824 (69.9%)
Late stages (stages B-D)	2789 (61.8%)	2502 (37.2%)	2094 (30.3%)	2075 (30.1%)
Surveillance efficiency, number of surveillance-detectedcases per 1000 surveillance tests	–	7.46	9.14	8.34
Low-risk	–	3.44	–	3.52
Intermediate-risk	–	6.42	6.42	6.69
High-risk	–	17.12	18.40	18.33

AFP, alpha fetoprotein; HCC, hepatocellular carcinoma; US, ultrasound.

### Cost-effectiveness analysis

Among all strategies, precision surveillance was the most costly ($10,312 million/100,000 population), followed by universal US + AFP ($10,211 million/100,000 population), risk-stratified surveillance ($10,124 million/100,000 population), and no surveillance ($7978 million/100,000 population) (Table 3). Precision surveillance generated the most QALYs (717,215 QALYs/100,000 population) compared to other strategies (714,943, 715,304, and 694,909 QALYs/100,000 population for universal US + AFP, risk-stratified, and no surveillance, respectively). Universal US + AFP and risk-stratified surveillance were both dominated, whereas precision surveillance was cost-effective with an ICER of $104,614/QALY gained, which was lower than the WTP threshold. Compared head-to-head with no surveillance, the ICERs of all three surveillance strategies were lower than the threshold of $150,000/QALY gained (universal US + AFP: $111,467/QALY gained; risk-stratified: $105,223/QALY gained; and precision: $104,614/QALY gained). If no surveillance was excluded from the analysis and universal US + AFP was the reference strategy, risk-stratified surveillance dominated universal US + AFP and was cost saving, meaning that it produced more QALYs at a lower cost than universal US + AFP. Comparing between precision and risk-stratified surveillance, the ICER of precision surveillance was $98,103/QALY gained, which was lower than the WTP threshold.

**Table 3:** Cost-effectiveness of surveillance strategies.

Surveillance strategy	Mean cost per 100,000 population (million $) [95% uncertainty interval]	Mean QALYs per 100,000 population [95% uncertainty interval]	ICER 1 (all strategies included)	ICER 2 (each strategy compared with no surveillance)	ICER 3 (no surveillance excluded)
No surveillance	$7978 [$7913–$8045]	694,909 [692,357–697,537]	Reference	Reference	–
Risk-stratified	$10,124 [$10,039–$10,209]	715,304 [712,698–717,965]	WD	$105,223	Cost saving
Universal US + AFP	$10,211 [$10,124–$10,295]	714,943 [712,302–717,561]	SD	$111,467	Reference; SD
Precision	$10,312 [$10,226–$10,395]	717,215 [714,685–719,800]	$104,614	$104,614	$98,103

AFP, alpha fetoprotein; ICER, Incremental cost effectiveness ratio; SD, strongly dominated; US, ultrasound; QALY, quality-adjusted life years; WD, weakly dominated.

– Strategy excluded from comparison.

Note: The 95% uncertainty intervals were calculated using the bootstrapping method.

Compared with universal US + AFP, risk-stratified and precision surveillance were more or equally efficient at detecting HCC cases. The screening efficiency (i.e., number of HCC cases/1000 tests) for risk-stratified and precision surveillance were 18.4 and 18.3 cases/1000 tests, respectively in high-risk patients, higher than that of universal US + AFP (17.1 cases/1000 tests). Compared to universal US + AFP, precision surveillance yielded more cases diagnosed at stage 0-A by identifying additional cases in intermediate- (+457 cases) and high-risk (+599 cases) patients, despite 273 fewer cases in low-risk patients (Figure S6). Compared to universal US + AFP, risk-stratified surveillance, detected fewer HCC cases at stage 0-A in low-risk patients, the same in intermediate-risk patients, and 591 more cases in high-risk patients.

### Sensitivity analyses

**Fig. 1:**
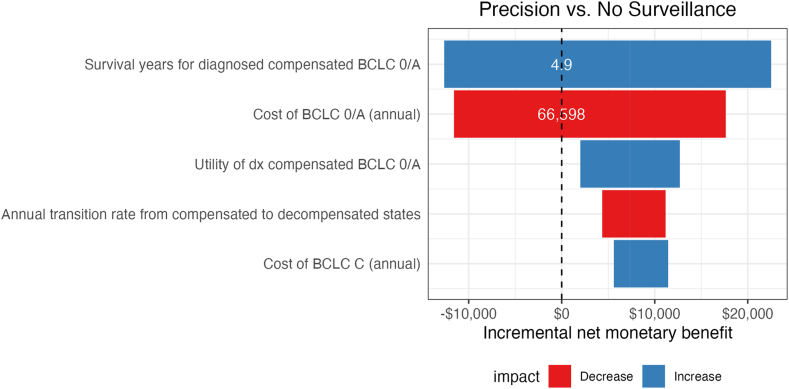
One-way sensitivity analysis. Tornado diagram with the top 5 parameters that most influence incremental net monetary benefits. Positive incremental net monetary benefit (NMB) indicates a higher NMB of precision surveillance compared to no surveillance. BCLC, Barcelona Clinic Liver Cancer Stage.

One-way sensitivity analyses were performed for all input parameters. Across all test sensitivities and specificities, precision surveillance consistently had a higher NMB than other strategies (Figure S7). Results were also consistent in a post-hoc sensitivity analysis in which the incremental utility of the diagnosed vs. undiagnosed state was varied from −0.1 to +0.1. The specificities of GALAD in male patients, obese patients, and those with viral liver disease were the top three parameters that had the greatest influence on NMB of precision surveillance compared to other strategies. Other than test performance, the parameters that most influenced incremental NMB are presented in Fig. 1. Notably, results were sensitive to the survival of early-stage HCC in patients with compensated cirrhosis and annual cost of treatment for early-stage HCC. Specifically, the optimal strategy changes from no surveillance to precision surveillance when survival of patients with compensated cirrhosis and early-stage HCC exceeded 4.9 years and annual costs of early-stage HCC were lower than $66,598.

**Fig. 2:**
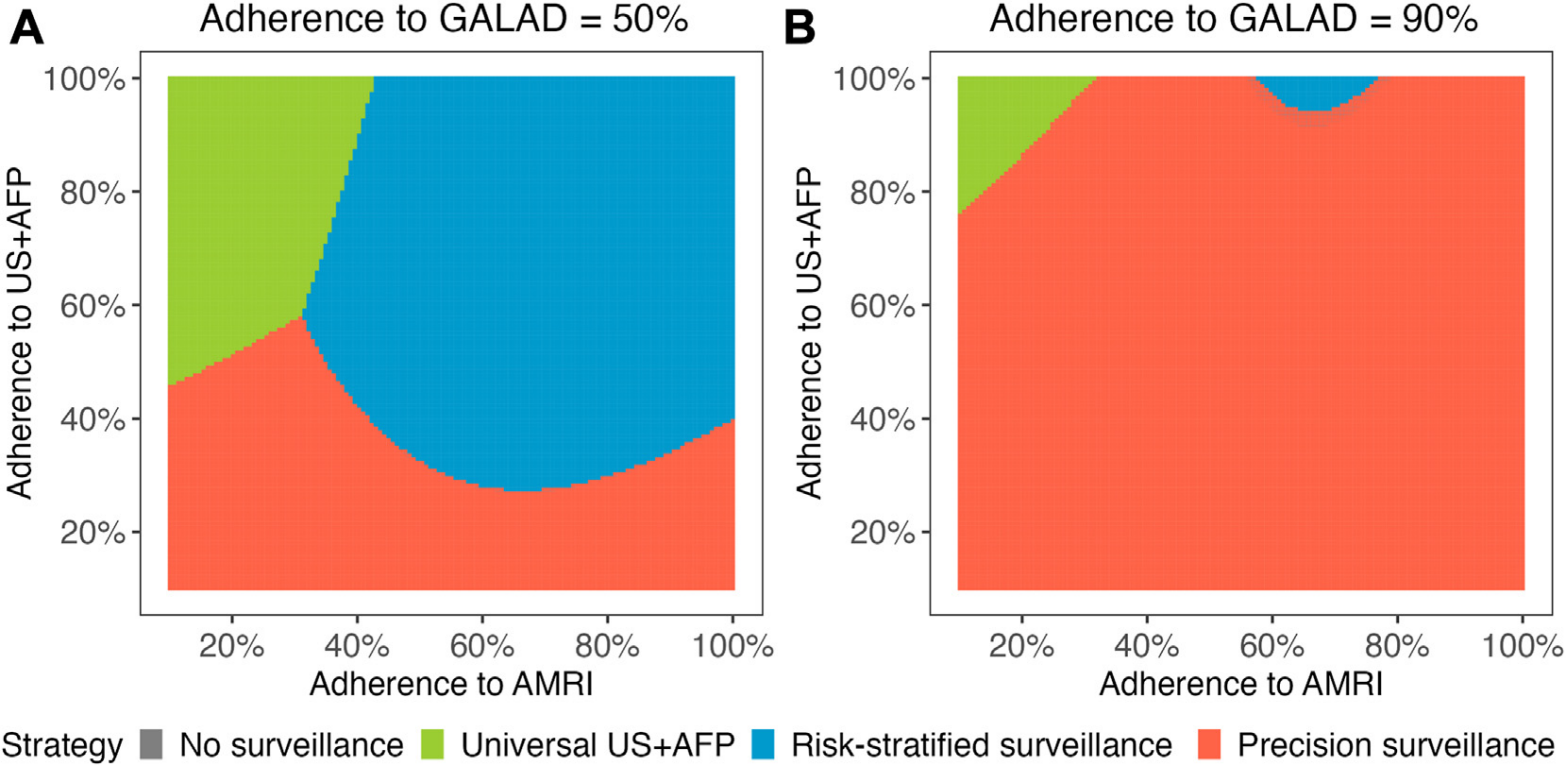
Three-way sensitivity analysis for adherence of surveillance modalities. The most cost-effective surveillance strategy was impacted by adherence to US + AFP, GALAD, and AMRI, albeit with non-linear associations. When the adherence for GALAD was 50%, precision surveillance outperformed the other surveillance strategies at lower adherence rates for US + AFP; however, universal US + AFP and risk-stratified surveillance were preferred when US + AFP and abbreviated MRI adherence increased. If adherence to GALAD was 90%, precision surveillance was the optimal strategy unless the adherence to US + AFP exceeded ∼80%. AFP, alpha fetoprotein; AMRI, abbreviated MRI; GALAD, Gender, Age, AFP-L3, AFP, and DCP; US, ultrasound.

Three-way sensitivity analysis showed that test-specific adherence had a non-linear influence on the optimal surveillance strategy (Fig. 2). When the adherence for GALAD was 50%, precision surveillance outperformed the other surveillance strategies at lower adherence rates for US + AFP and AMRI; however, universal US + AFP and risk-stratified surveillance could be optimal as US + AFP adherence increased. If adherence to GALAD increased to 90%, precision surveillance was the optimal strategy unless the adherence to US + AFP exceeded ∼80%. In a hypothetical scenario with adherence estimates of 40% for US + AFP (based on published literature), lower adherence for AMRI of 30% (given increased logistical barriers), and higher adherence for GALAD at 55% (given decreased barriers), precision surveillance was a cost-effective strategy with and ICER of $110,525 (Table S5).

**Fig. 3:**
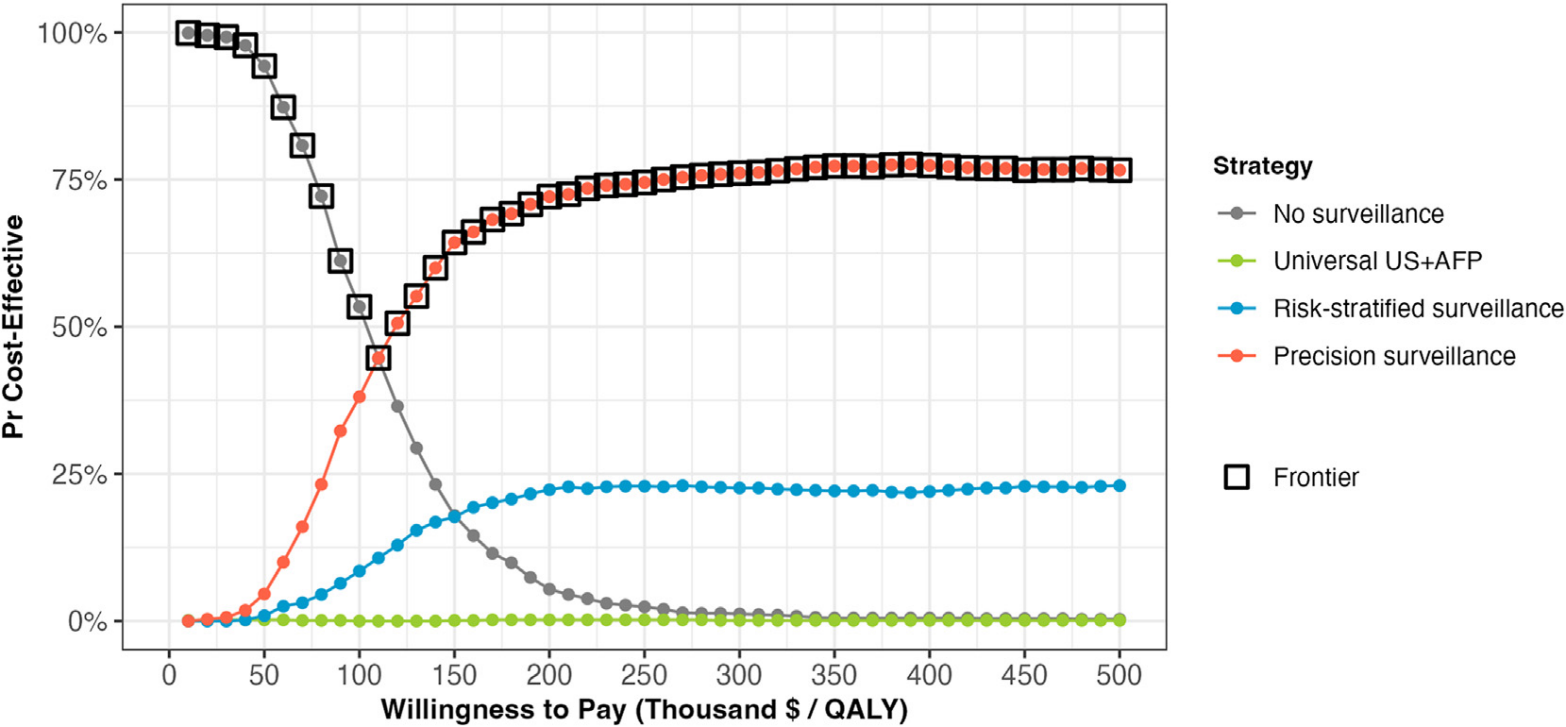
Cost-effectiveness acceptability curve. Across 1000 probabilistic sample analyses, precision surveillance became the most likely strategy to be cost-effective at willingness-to-pay thresholds exceeding $110,000/QALY gained. Universal US + AFP was cost-effective in less than 1% of PSA samples across all WTP thresholds. AFP, alpha fetoprotein; US, ultrasound.

The CEAC is presented in Fig. 3. Across 1000 PSA samples, no surveillance was most likely to be cost-effective at thresholds <$110,000/QALY gained, while precision surveillance became the most likely to be cost-effective at WTP thresholds exceeding $110,000/QALY gained. Universal US + AFP was cost-effective in less than 1% of PSA samples across all WTP thresholds, whereas risk-stratified surveillance was more likely to be cost-effective than no surveillance at thresholds >$150,000/QALY in 20% of PSA samples. For the CEAC of head-to head comparison with no surveillance (Figure S8), universal US + AFP was likely to be cost-effective at thresholds >$130,000/QALY, and both precision and risk-stratified surveillance became more likely to be cost-effective at thresholds >$110,000/QALY.

## Discussion

Current paradigms of HCC surveillance consist of a one-size-fits-all approach, with recommendations for ultrasound plus AFP in all patients despite variable test performance and risk of HCC based on demographic and disease specific factors.^3^ We conducted a cost-effectiveness analysis of various personalized surveillance, including risk stratified surveillance in which individual risk was accounted for and precision surveillance in which test performance and individual risk were both included. We found that precision surveillance yielded the most cost-effective strategy compared to universal ultrasound plus AFP. The precision strategy had the highest likelihood of early-stage diagnosis, which translated into a survival benefit and cost-effectiveness.

Our study is the first cost-effectiveness model to examine the cost-effectiveness of personalized HCC surveillance strategies. Prior models have found that universal ultrasound plus AFP is a cost-effective strategy, while universal CT or MRI based surveillance do not meet thresholds for cost-effectiveness.^17^^,^^61^^,^^62^ Compared to prior models, our results using updated inputs suggests continued advances in locoregional and systemic therapies may mitigate survival differences of early-stage detection and the cost-effectiveness of universal ultrasound plus AFP surveillance. We incorporated several novel surveillance methods into our model, including blood-based surveillance with GALAD and imaging-based surveillance with abbreviated MRI. While these surveillance methodologies are currently undergoing more rigorous evaluation, they both are considered highly promising strategies based on available validation data and patient preferences.^16^^,^^32^^,^^63^

Our estimates of individual risk were based on blood-based and clinical risk prediction models; however, these require further validation and calibration with implementation in clinical practice to execute a precision surveillance strategy. Implementation of precision surveillance could present several logistical difficulties, including provider and patient confusion as to what test to complete, patient access to some surveillance modalities, such as abbreviated MRI, and acceptability of no surveillance in low-risk individuals. These difficulties could in part be overcome through robust decision support tied to the electronic medical record and patient and provider education. Indeed, a precision surveillance paradigm could exacerbate health disparities given its complexity and access to certain strategies such as abbreviated MRI. Robust automated measurement mechanisms for surveillance adherence would be necessary to monitor for emergence of these disparities. On the other hand, focused intensity of surveillance on the highest risk individuals could allow for concentrated efforts of increasing adherence in those populations.^64^^,^^65^ Given these logistical difficulties, a risk-stratified approach may be preferable in some settings, as our analysis also cost saving compared to the current approach using universal ultrasound plus AFP.

Additionally, we note that risk-stratified and precision surveillance strategies can miss early-stage HCC in low-risk patients compared to universal ultrasound plus AFP. Although this shortcoming is offset by increased early-stage detection in intermediate- and high-risk subgroups, these data highlight a need for improved risk stratification in the future. Although providers are generally accepting of risk-based surveillance strategies, large numbers of missed cancers may reduce adoption of a no-surveillance strategy in low-risk patients.

There are several strengths and limitations of this manuscript that warrant further attention. One limitation is that our model assumes stable HCC risk over time, but HCC risk can be dynamic as patients age and patients could transition to a higher or lower risk state with aging or disease modifying interventions such as treatment of viral hepatitis, weight loss, or alcohol cessation.^66^ Similarly, the optimal surveillance strategy for an individual patient may also change over time. Second, as noted, some surveillance methods (i.e., abbreviated MRI and GALAD) have emerging data on test performance in a surveillance setting and the estimates used for this analysis may not reflect real world performance. Third, we developed the precision surveillance strategy based on preferred strategies for each patient profile, although a more cost-effective overall combination may be possible in the future. Fourth, our precision surveillance strategy included ∼50 different patient profiles, although a more nuanced precision surveillance strategy may be possible by differentiating factors further (e.g., ALD vs. MASLD). However, optimizing performance of a surveillance strategy must be balanced with feasibility of implementation in clinical practice. Although decision support tools in the electronic medical record would have minimal impact on costs, other interventions for implementation and adherence could impact cost-effectiveness of the precision surveillance strategy. Finally, there are several emerging modalities for HCC surveillance, e.g., cell free DNA methods; however, these strategies are in early-phase validation and thus not included.^10^ These limitations are outweighed by the strengths of the novelty of our analysis, nuanced modeling methodology, and high clinical implications of our findings.

In conclusion, we showed that a precision surveillance strategy is the most cost-effective method for HCC surveillance. This approach could maximize surveillance benefits, while minimizing harms associated with surveillance, although further studies evaluating the impact on downstream outcomes would be necessary. While implementation of such a surveillance paradigm may be challenging, there could be marked improvements of the effectiveness of existing HCC surveillance programs.

## Contributors

The authors confirm contribution to the paper as follows:

Study conception and design: SZK, KS, NDP, AGS.

Data collection: NF, YH, NDP, AGS.

Analysis and interpretation of results: SZK, KS, NDP, AGS.

Draft manuscript preparation: SZK, KS, NDP, AGS.

All authors reviewed the results and approved the final version of the manuscript.

## Data sharing statement

The authors confirm that data supporting the findings of the study are available within the article and its supplemental material. No additional new data were generated in this study.

## Declaration of interests

Amit G. Singal has served as a consultant or on advisory boards for Genentech, AztraZeneca, Eisai, Bayer, Exelixis, Merck, Boston Scientific, Sirtex, HistoSonics, FujiFilm Medical Sciences, Exact Sciences, Roche, Glycotest, Freenome, and GRAIL.

Neehar D. Parikh has served as a consultant or advisory boards for Eisai, Exelixis, Fujifilm Medical Sciences, and Gilead.

Yujin Hoshida. advisory: Helio Genomics, Espervita Therapeutics, Alentis Therapeutics, Roche Diagnostics, Elevar Therapeutics; shareholder: Espervita Therapeutics, Alentis Therapeutics.

Szu-Yu Zoe Kao is employed by Siemens Medical Solutions USA, Inc.

Kinpritma Sangha is employed by and owns stock in Siemens Medical Solutions USA, Inc.
