# Supplementary material for: Cost-effectiveness of a precision hepatocellular carcinoma surveillance strategy in patients with cirrhosis
Source: eClinicalMedicine. 2024 Aug 13;75:102755. doi: 10.1016/j.eclinm.2024.102755 (PMC11372615; doi:10.1016/j.eclinm.2024.102755)
Supplement: Supplementary Figures and Tables [file mmc1.docx]

Supplemental Materials Table of Contents

[SUPPLEMENTARY METHODS 2](#_Toc169006186)

[**Stage-specific annual incidental detection rate** 2](#_Toc169006187)

[**Survival for HCC stages by diagnosis and cirrhosis decompensation status** 3](#_Toc169006188)

[**Developing precision surveillance** 3](#_Toc169006189)

[**Test sensitivity and specificity by patient risk factor** 4](#_Toc169006190)

[SUPPLEMENTARY TABLES 8](#_Toc169006191)

[**Table S1** 8](#_Toc169006192)

[**Table S2** 9](#_Toc169006193)

[**Table S3** 10](#_Toc169006194)

[**Table S4** 12](#_Toc169006195)

[**Table S5** 14](#_Toc169006195)

[SUPPLEMENTARY FIGURES 15](#_Toc169006196)

[**Figure S1.** 16](#_Toc169006197)

[**Figure S2.** 16](#_Toc169006197)

[**Figure S3.** 17](#_Toc169006198)

[**Figure S4.** 18](#_Toc169006199)

[**Figure S5.** 19](#_Toc169006200)

[**Figure S6.** 20](#_Toc169006201)

[**Figure S7.** 21](#_Toc169006202)

[**Figure S8.** 22](#_Toc169006203)

[SUPPLEMENTARY REFERENCES 21](#_Toc169006204)

# **SUPPLEMENTARY METHODS**

## **Stage-specific annual incidental detection rate**

The monthly probability of incidental detection was likely to vary by disease severity. However, these parameters were not estimated in the existing literature. We estimated the monthly probability of incidental detection for stage 0-A via calibration to hospital data and derived the monthly probability of incidental detection for stages B-D (i.e., later stages) via assumption for stage-specific survival estimates. We did not calibrate the monthly probability of incidental detection for later stages as did for stage 0-A because of the small number of observations of patients with these disease states (<100 observations) in the hospital data in the U.S.^1^ For HCC stages B-D, the annual incidental detection rate of a disease state was the inverse of the median survival estimate of the corresponding undiagnosed disease state in **Table S2**. This calculation allowed the incidental detection rates for later stages to be slightly higher than the death rate by disease state, meaning that on average patients in these undiagnosed disease states could develop symptoms before they died. The annual rates were converted to monthly rate and then to monthly probability using the declining exponential approximation.^1^

Regarding monthly probability of incidental detection at stage 0-A, we obtained the data on the cases detected at stage 0-A from four health systems in the U.S.^2^ In 2012-2013, these health systems reported that 79 out of 178 patients (44.4%: calibration target) diagnosed with stage 0-A were *not* detected through surveillance. To calibrate the monthly probability of incidental detection at stage 0-A, we assumed the population received ultrasound-based surveillance (ultrasound plus alpha fetoprotein: US+AFP) at a surveillance utilization rate of 40% every 6 months,^3, 4^ allowing HCC cases to be detected via surveillance. In addition to surveillance-detected cases, individuals with HCC could be detected incidentally at a stage-specific monthly probability, which was derived above. Calibration was performed using grid search algorithm.

The derived and calibrated monthly probabilities of incidental detection are presented in **Table S1**. Based on the estimated probabilities of incidental detection, our model generated that 44.8% of the HCC cases diagnosed at stage A were incidentally detected.

## **Survival for HCC stages by diagnosis and cirrhosis decompensation status**

Median survival for decompensated cirrhosis and median HCC stage-specific survival by diagnosis status were estimated from the literature (**Table S2**).^5-8^ However, data on stage-specific survival by both diagnosis and cirrhosis decompensation status were lacking. Therefore, to derive more detailed survival parameters, we used the minimum survival estimate for a disease state across all the relevant survival estimates to that disease state. For example, the median survival for a patient with diagnosed decompensated HCC stage A was 2.5 years, which is the minimum survival between decompensated cirrhosis (2.5 years) and diagnosed stage A (8 years).

## **Developing precision surveillance**

We employed the cost-effectiveness analytical framework to develop the patient-type-specific surveillance strategies in precision screening by the following steps. First, we derived risk categories using clinical risk stratification models that are widely available, have been used in prior studies, and implemented in some health systems.^9-11^ Second, we simulated 100,000 patients for a patient type of interest (e.g., low-risk obese male patients with viral cirrhosis and Child-Pugh A) to obtain the patient type-specific lifetime costs and QALYs by US+AFP, AMRI, GALAD, and no surveillance, respectively. Third, the surveillance strategies were sorted by the costs in increasing order for a type of patient. A surveillance strategy with QALYs fewer than the previous less costly strategy was strongly dominated and eliminated from the comparison. Fourth, the incremental costs and QALYs for each testing strategy were calculated as the difference in costs and QALYs between the strategy of interest and the previous less costly strategy. The incremental cost-effectiveness ratio (ICER) for a testing strategy was a ratio of the incremental costs to the incremental QALYs. Fifth, weakly dominated testing strategies, which had ICERs higher than the next costly strategy, were eliminated from the comparison. ICERs were recalculated among the remaining strategies. Finally, we compared the ICER of each surveillance strategy with a willingness-to-pay (WTP) threshold of $150,000/QALY gained, which was used to account for the recent trend of inflation in the U.S.^12, 13^ The most cost-effective strategy for a patient type was the strategy with the largest ICER lower than the threshold. We repeated this process for all 48 patient types.

## **Test sensitivity and specificity by patient risk factor**

We considered risk factors that can affect test performance in this study. Four dichotomous risk factors were considered: sex (male vs. female), etiology (viral vs. non-viral), Child-Pugh class (class A vs. B/C), and body weight (obese vs. non-obese). With all patient risk factors combined, there were 16 types of patients. It is worth noting that the number of patient types is different to that in the main text (48 patient types) because the three risk groups were not accounted in deriving test sensitivity and specificity.

We derived a test sensitivity and specificity estimate for each type of patient by US+AFP, AMRI, and GALAD. However, test sensitivity and specificity were only reported as a marginal estimate for each category of a risk factor in the literature, instead of a joint estimate that accounts all patient risk factors.^14-16^ To derive a test sensitivity and specificity simultaneously accounted for all four risk factors, we first obtained or derived the marginal estimate for each category of a risk factor for a surveillance modality, giving rise to four estimates for each type of patients (**Table S4**). For each type of patients, the minimum among all four marginal estimates associated with the type of patients was used as the final sensitivity or specificity in the simulation model.

*Marginal test sensitivity by patient risk factor*

Test sensitivity was estimated by the types of patients for early stage (stage A) and late stages (stages B-C), respectively following the steps described above.

For AMRI and GALAD, studies reported the marginal test sensitivity for stage A for each risk factor by category.^15, 16^ However, categories of a risk factor defined in these studies were sometimes different (e.g., sensitivity reported for normal weight, obese, and overweight separately) to those in our study. In the cases where discrepancy occurred, a weighted estimate was derived. For example, the sensitivity of AMRI for stage A among patients with non-viral liver diseases was 87.6%. This sensitivity was weighted among three sensitivity estimates, 82.1% for patients with alcohol liver diseases (47% of patients), 97.2% for patients with non-alcoholic fatty liver diseases (35% of patients), and 83.3% for patients with other liver diseases (19% of patients) as reported in Yokoo et al.^15^

For the sensitivity of US+AFP for stage A, we used the odds ratios reported for the risk factors associated with worse surveillance quality from Schoenberger et al.^17^ to inform the differences in test sensitivity for each risk factor by category. Schoenberger et al. did not provide the reference sensitivity of US+AFP for each risk factor. Therefore, we assumed that the overall sensitivity of US+AFP for stage A was 63% and treated this as the grand mean between categories of a risk factor.^14^ The marginal sensitivity for stage A of a category of a risk factor, $\mathrm{Sens}\left( x_{i} \right),$ was derived by borrowing the concept of contrast coding from statistical literature.^18^ First, the grand mean was converted to log odds ($\alpha$). Next, the odds ratio ($\beta_{x}$) for a risk factor *x* reported in Schoenberger et al was log transformed. All the odds ratios were inversed because these risk factors negatively impacted the test performance compared with the reference category. The marginal sensitivity for stage A of a risk category was estimated in equation (1).

$$\begin{aligned} \mathrm{Sens}\left( x_{i} \right)=\left\{ \begin{aligned} \frac{\mathrm{Exp}\left( \alpha-0.5\cdot\beta_{x} \right)}{1+Exp\left( \alpha-0.5\cdot\beta_{x} \right)} , i=0 \\ \frac{\mathrm{Exp}\left( \alpha+0.5\cdot\beta_{x} \right)}{1+Exp\left( \alpha+0.5\cdot\beta_{x} \right)}, i=1 \end{aligned} \right. \#\left( 1 \right) \end{aligned}$$

Where *i* indicates whether the category is the reference case (=0) or not (=1); $x_{i}$ represents category *i* of risk factor *x*. Following these steps, the sensitivity of US+AFP for stage A was 64% for males and 62% for females. We repeated the calculation for other risk factors to obtain the marginal estimates.

For US+AFP and GALAD, the sensitivity for later stages by category of a risk factor was calculated as follows. We obtained the overall sensitivity for later stages for each test modality,^14, 16^ and calculated the log of odds ratio ($\beta'$) of the overall sensitivity of later stages to the overall sensitivity of stage A. The log of odds ratio served as the slope in the logistic function. In addition, the test sensitivity for stage A estimated for a risk category was converted to log scale (${\alpha'}_{x}$), which was regarded as the intercept in the logistic function. The test sensitivity for later stages for a category of a risk factor, $Sens'\left( {x'}_{i} \right)$, was derived in equation (2).

$$\begin{aligned} Sens'\left( {x'}_{i} \right)=\frac{\mathrm{Exp}\left( \alpha_{x}^{'}+\beta^{'} \right)}{1+Exp\left( {\alpha^{'}}_{x}+\beta^{'} \right)} \#\left( 2 \right) \end{aligned}$$

For AMRI, we employed a different method to calculate the sensitivity for later stages because the method used for US+AFP and GALAD yielded a wide variation of the estimates of AMRI, which does not reflect the observation in clinical practice. We used the overall sensitivity for later stages for AMRI to be similar to US+AFP and allowed the estimate for each risk category to deviate 1-2 percentage points from the overall estimate.

*Marginal specificity by patient risk factor*

The marginal specificity for AMRI by category of each risk factor was obtained directly from Yokoo et al.^15^ Weighted average was applied as did for test sensitivity. For US+AFP, studies have shown a lower ultrasound quality associated with non-viral cirrhosis, Child-Pugh B/C, and obesity.^19^ Thus, we assumed the specificity as follows: 86% for viral cirrhosis vs. 82% for non-viral cirrhosis; 88% for Child-Pugh A vs. 80% for B/C; 86% for non-obese vs. 82% for obese patients; no differences between males and females (84%).^19, 20^ For GALAD, we assumed that the overall specificity was 86% and the specificity only differed between viral (80%) and non-viral (88%) liver disease.^16, 21^

*Deriving test sensitivity and specificity accounting for all patient risk factors*

To obtain the test sensitivity and specificity for a type of patient (e.g., obese males with viral cirrhosis and Child-Pugh class A), we used the minimum estimate among all four marginal estimates associated with the specific patient type. This process was repeated for each type of patient by US+AFP, AMRI, and GALAD, respectively. The final test sensitivity and specificity estimates used in the simulation model is visualized in **Figure S2**.

# **SUPPLEMENTARY TABLES**

**Table S1**. The monthly incidental detection rate for decompensated cirrhosis and by HCC stage.

| **Disease state at diagnosis** | **Median survival time for undiagnosed state** | **Monthly incidental detection rate** | **Source** |
| --- | --- | --- | --- |
| BCLC stage A |  | 2.5% | Calibrated |
| BCLC stage B | 1.5 years | 5.4% | Assumption, calculated |
| BCLC stage C | 0.75 years | 10.5% | Assumption, calculated |
| BCLC stage D | 0.5 years | 15.4% | Assumption, calculated |

**Table S2**. Median years of overall survival by decompensated cirrhosis, HCC stage, and diagnosis status.

| **Disease state** | **Median overall survival (years)** | **Range** |
| --- | --- | --- |
| Decompensated cirrhosis | 2.5 | 0.5–5.0 |
| BCLC stage A |  |  |
| Undiagnosed | 2.9 | 1.0–4.0 |
| Diagnosed | 8 | 1.0–19.0 |
| BCLC stage B |  |  |
| Undiagnosed | 1.5 | 0.0–3.0 |
| Diagnosed | 3.7 | 0.0–8.5 |
| BCLC stage C |  |  |
| Undiagnosed | 0.75 | 0.5–1.2 |
| Diagnosed | 1.5 | 1.0–3.0 |
| BCLC stage D (diagnosed/undiagnosed) | 0.5 | 0.25–0.75 |

**Table S3**. Population distribution of patient types in cirrhosis patients.

| **Patient characteristics** | | | | |  |
| --- | --- | --- | --- | --- | --- |
| **Risk group** | **Sex** | **Body weight** | **Child-Pugh class** | **Etiology** | **Population weight (%)** |
| Low | Female | Not obese | Child A | Viral | 1.15% |
| Low | Female | Not obese | Child A | Non-viral | 2.78% |
| Low | Female | Not obese | Child B/C | Viral | 0.19% |
| Low | Female | Not obese | Child B/C | Non-viral | 1.49% |
| Low | Female | Obese | Child A | Viral | 1.15% |
| Low | Female | Obese | Child A | Non-viral | 2.98% |
| Low | Female | Obese | Child B/C | Viral | 0.14% |
| Low | Female | Obese | Child B/C | Non-viral | 0.53% |
| Low | Male | Not obese | Child A | Viral | 3.70% |
| Low | Male | Not obese | Child A | Non-viral | 2.21% |
| Low | Male | Not obese | Child B/C | Viral | 0.14% |
| Low | Male | Not obese | Child B/C | Non-viral | 0.96% |
| Low | Male | Obese | Child A | Viral | 2.54% |
| Low | Male | Obese | Child A | Non-viral | 2.64% |
| Low | Male | Obese | Child B/C | Viral | 0.19% |
| Low | Male | Obese | Child B/C | Non-viral | 0.43% |
| Intermediate | Female | Not obese | Child A | Viral | 1.63% |
| Intermediate | Female | Not obese | Child A | Non-viral | 3.60% |
| Intermediate | Female | Not obese | Child B/C | Viral | 0.91% |
| Intermediate | Female | Not obese | Child B/C | Non-viral | 3.07% |
| Intermediate | Female | Obese | Child A | Viral | 1.97% |
| Intermediate | Female | Obese | Child A | Non-viral | 4.99% |
| Intermediate | Female | Obese | Child B/C | Viral | 0.82% |
| Intermediate | Female | Obese | Child B/C | Non-viral | 3.31% |
| Intermediate | Male | Not obese | Child A | Viral | 6.57% |
| Intermediate | Male | Not obese | Child A | Non-viral | 4.94% |
| Intermediate | Male | Not obese | Child B/C | Viral | 1.68% |
| Intermediate | Male | Not obese | Child B/C | Non-viral | 4.61% |
| Intermediate | Male | Obese | Child A | Viral | 5.90% |
| Intermediate | Male | Obese | Child A | Non-viral | 6.77% |
| Intermediate | Male | Obese | Child B/C | Viral | 1.49% |
| Intermediate | Male | Obese | Child B/C | Non-viral | 3.46% |
| High | Female | Not obese | Child A | Viral | 0.67% |
| High | Female | Not obese | Child A | Non-viral | 0.48% |
| High | Female | Not obese | Child B/C | Viral | 0.29% |
| High | Female | Not obese | Child B/C | Non-viral | 1.49% |
| High | Female | Obese | Child A | Viral | 0.67% |
| High | Female | Obese | Child A | Non-viral | 0.86% |
| High | Female | Obese | Child B/C | Viral | 0.58% |
| High | Female | Obese | Child B/C | Non-viral | 1.63% |
| High | Male | Not obese | Child A | Viral | 1.87% |
| High | Male | Not obese | Child A | Non-viral | 0.77% |
| High | Male | Not obese | Child B/C | Viral | 1.44% |
| High | Male | Not obese | Child B/C | Non-viral | 2.45% |
| High | Male | Obese | Child A | Viral | 1.78% |
| High | Male | Obese | Child A | Non-viral | 1.06% |
| High | Male | Obese | Child B/C | Viral | 1.63% |
| High | Male | Obese | Child B/C | Non-viral | 3.41% |

**Table S4**. The marginal estimate of test sensitivity and specificity by patient risk factors between early stage (stage A) and late stages (stages B-D) for US+AFP, AMRI, and biomarker (GALAD), respectively.

|  | **Marginal estimate of test sensitivity and specificity** | | |
| --- | --- | --- | --- |
| **Category** | **US+AFP^†^** | **AMRI** | **Biomarker (GALAD)** |
| Sensitivity [Range] | | | |
| *BCLC stage A* |  |  |  |
| Overall | 63% [50%–80%] | 88% [60%–99%] | 67% [45%–80%] |
| Sex |  |  |  |
| Female | 62% [50%–80%] | 83% [60%–99%] | 50% [45%–80%] |
| Male | 64% [50%–80%] | 90% [60%–99%] | 75% [45%–80%] |
| Body weight |  |  |  |
| Not obese | 73% [50%–80%] | 87% [60%–99%] | 72% [45%–80%] |
| Obese | 52% [50%–80%] | 91% [60%–99%] | 62% [45%–80%] |
| Child-Pugh class |  |  |  |
| A | 67% [50%–80%] | 94% [60%–99%] | 67% [45%–80%] |
| B/C | 59% [50%–80%] | 64% [60%–99%] | 73% [45%–80%] |
| Etiology |  |  |  |
| Viral | 70% [50%–80%] | 88% [60%–99%] | 68% [45%–80%] |
| Non-viral | 55% [50%–80%] | 88% [60%–99%] | 72% [45%–80%] |
| *BCLC stages B-D* |  |  |  |
| Overall | 97% [90%–99%] | 97% [80%–99%] | 91% [80%–99%] |
| Sex |  |  |  |
| Female | 97% [90%–99%] | 96% [80%–99%] | 83% [80%–99%] |
| Male | 97% [90%–99%] | 98% [80%–99%] | 94% [80%–99%] |
| Body weight |  |  |  |
| Not obese | 98% [90%–99%] | 96% [80%–99%] | 93% [80%–99%] |
| Obese | 95% [90%–99%] | 89% [80%–99%] | 89% [80%–99%] |
| Child-Pugh class |  |  |  |
| A | 97% [90%–99%] | 99% [80%–99%] | 91% [80%–99%] |
| B/C | 96% [90%–99%] | 95% [80%–99%] | 93% [80%–99%] |
| Etiology |  |  |  |
| Viral | 98% [90%–99%] | 97% [80%–99%] | 92% [80%–99%] |
| Non-viral | 96% [90%–99%] | 97% [80%–99%] | 93% [80%–99%] |
| Specificity [Range] | | | |
| Sex |  |  |  |
| Female | 84% [75%–95%] | 86% [60%–99%] | 86% [75%–95%] |
| Male | 84% [75%–95%] | 91% [60%–99%] | 86% [75%–95%] |
| Body weight |  |  |  |
| Not obese | 86% [75%–95%] | 88% [60%–99%] | 86% [75%–95%] |
| Obese | 82% [75%–95%] | 91% [60%–99%] | 86% [75%–95%] |
| Child-Pugh class |  |  |  |
| A | 88% [75%–95%] | 67% [60%–99%] | 86% [75%–95%] |
| B/C | 79% [75%–95%] | 90% [60%–99%] | 86% [75%–95%] |
| Etiology |  |  |  |
| Viral | 86% [75%–95%] | 79% [60%–99%] | 80% [75%–95%] |
| Non-viral | 82% [75%–95%] | 95% [60%–99%] | 88% [75%–95%] |
| *Note:* The ranges of parameters are used in one-way sensitivity and probabilistic sensitivity analyses (PSA). In PSA, all test sensitivities and specificities are assumed to follow the PERT distribution, specified as PERT(min=lower bound, mode=mean, max=upper bound, shape=4).  ^†^ The overall sensitivity estimates for US+AFP was estimated in Tzartzeva et al. The marginal sensitivity estimates were calculated based on the overall estimates and the odds ratios reported in Schoenberger et al. | | | |

**Table S5**. Cost-effectiveness of surveillance strategies with hypothetical estimates of surveillance non-adherence

| **Surveillance strategy** | **Mean cost per 100,000 population  (million $)**  **[95% uncertainty interval]** | **Mean QALYs per 100,000 population**  **[95% uncertainty interval]** | **ICER 1 (all strategies included)** | **ICER 2 (each strategy compared with no surveillance)** | **ICER 3 (no surveillance excluded)** |
| --- | --- | --- | --- | --- | --- |
| No surveillance | $7,978  [$7,905–$8,047] | 694,909  [692,270–697,529] | Reference | Reference | — |
| Risk-stratified | $9,031  [$8,953–$9,107] | \| 704,816 \| \| --- \|   [702,211–707,436] | WD | $106,266 | Reference |
| Universal US+AFP | $9,174  [$9,095–$9,249] | 705,306  [702,704–707,807] | WD | $115,001 | WD |
| Precision | \| $9,325 \| \| --- \|   [$9,244–$9,404] | \| 707,099 \| \| --- \|   [704,485–709,629] | $110,525 | $110,525 | $129,004 |
| Abbreviation: QALY = quality-adjusted life years; SD = strongly dominated; WD = weakly dominated — Strategy excluded from comparison  Note: The 95% uncertainty intervals were calculated using the bootstrapping method. | | | | | |

**SUPPLEMENTARY FIGURES**

**Figure S1.** State-transition diagram of natural disease progression of HCC from compensated cirrhosis, to decompensated cirrhosis, HCC stages (0/A-D).


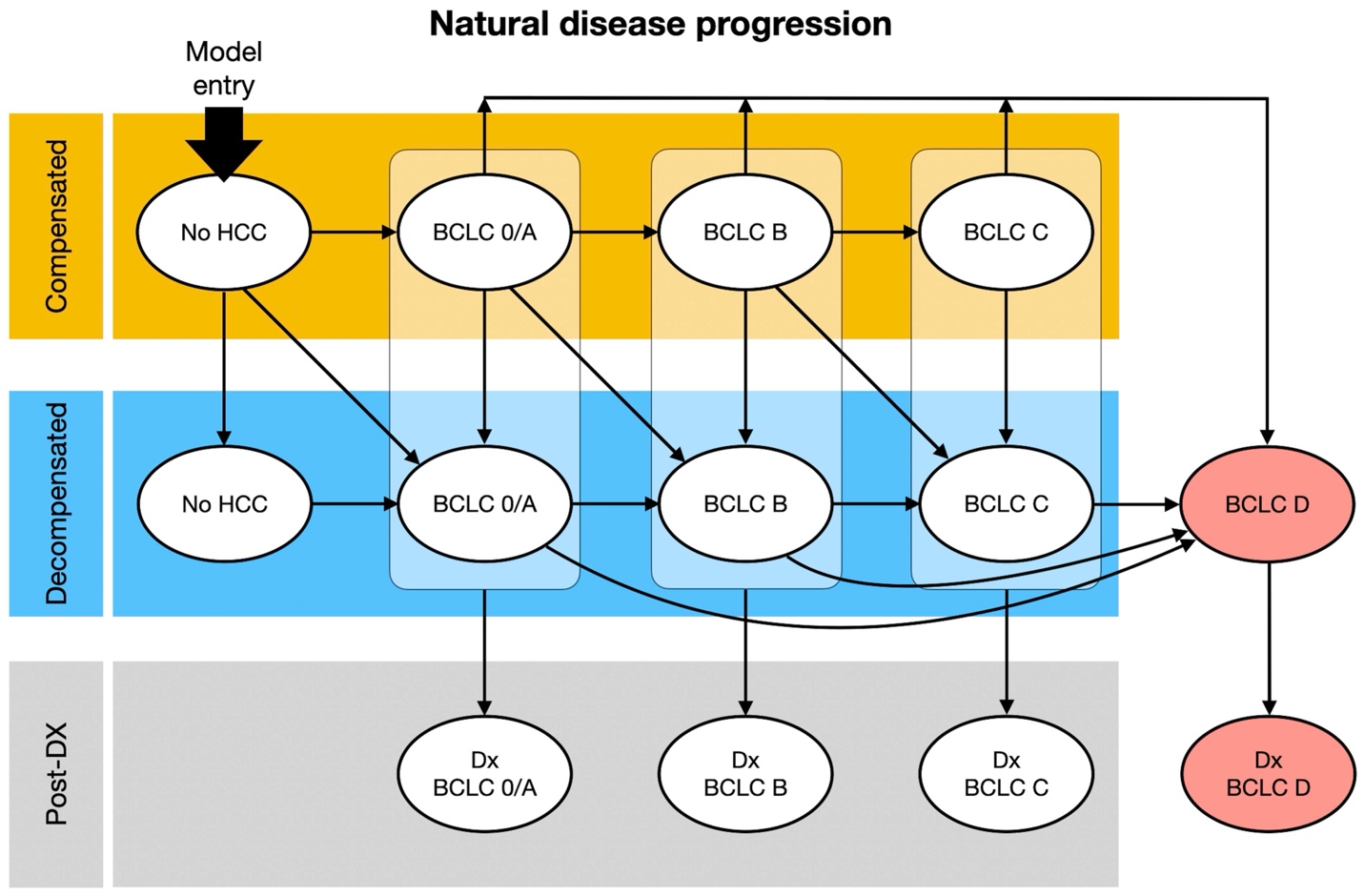


**Figure S2.** Test sensitivity and specificity by surveillance modality, HCC stage, and patient risk factors

**
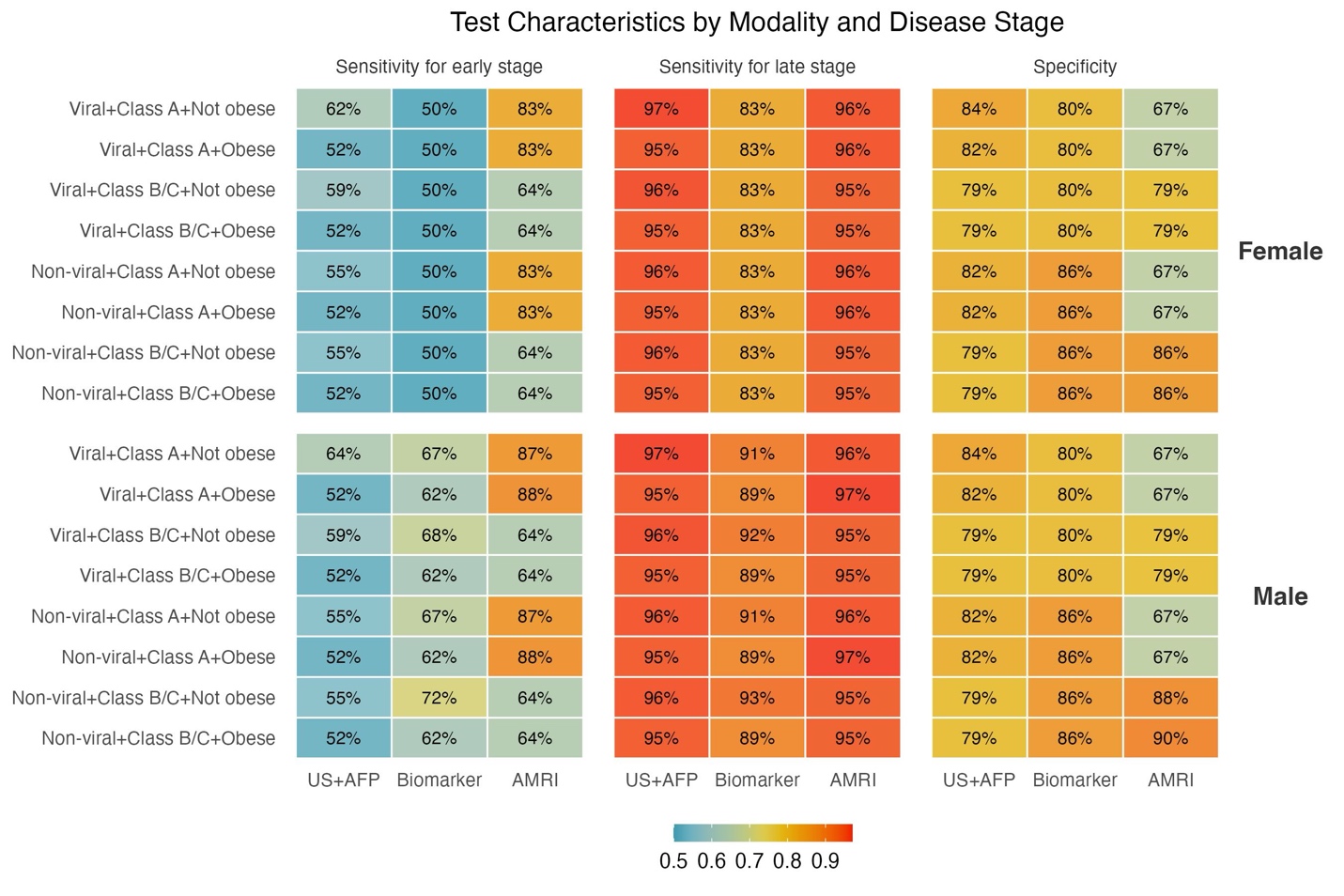
**

Early stage indicates BCLC stage 0-A and late stage indicates BCLC stages B-D.

**Figure S3.** Patient surveillance pathway following semiannual surveillance.


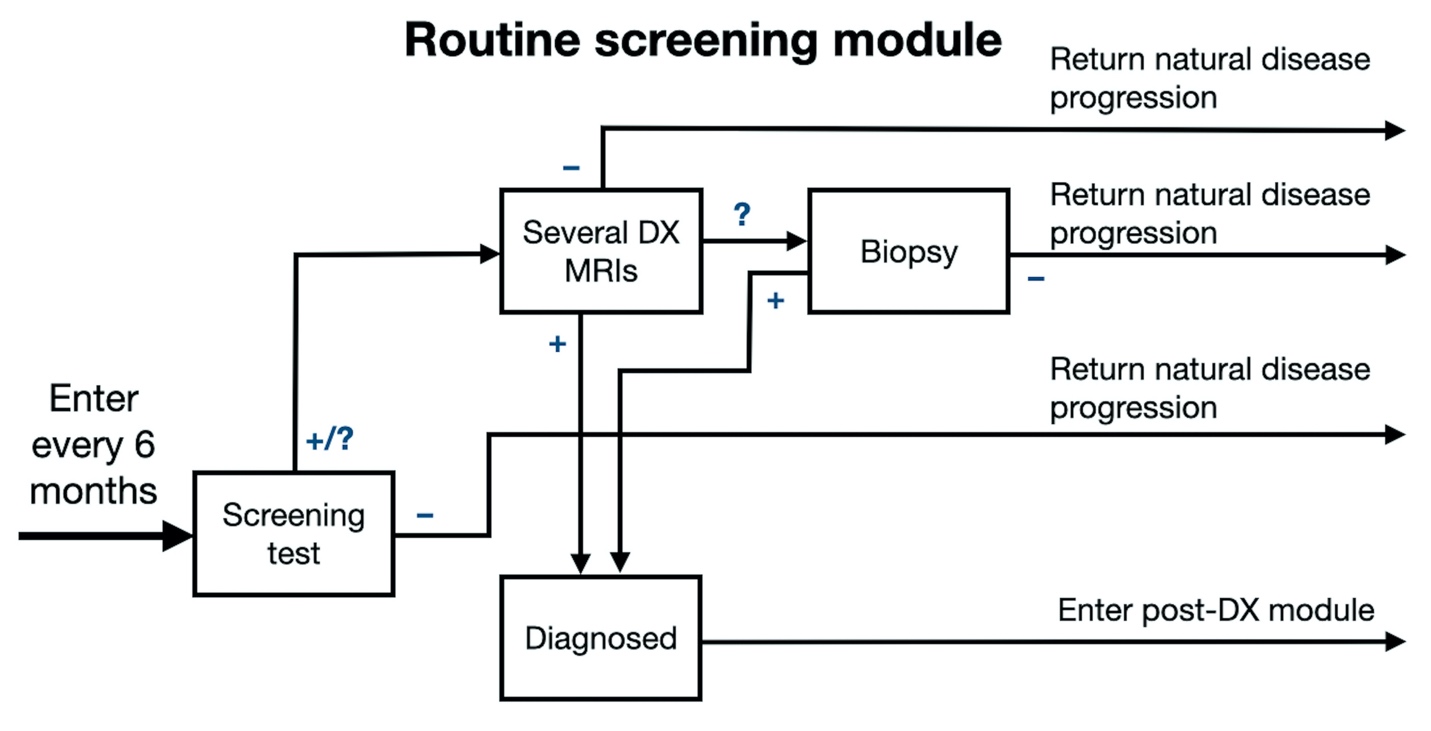


**Figure S4.** Testing strategy by patient type in precision and risk-stratified surveillance.


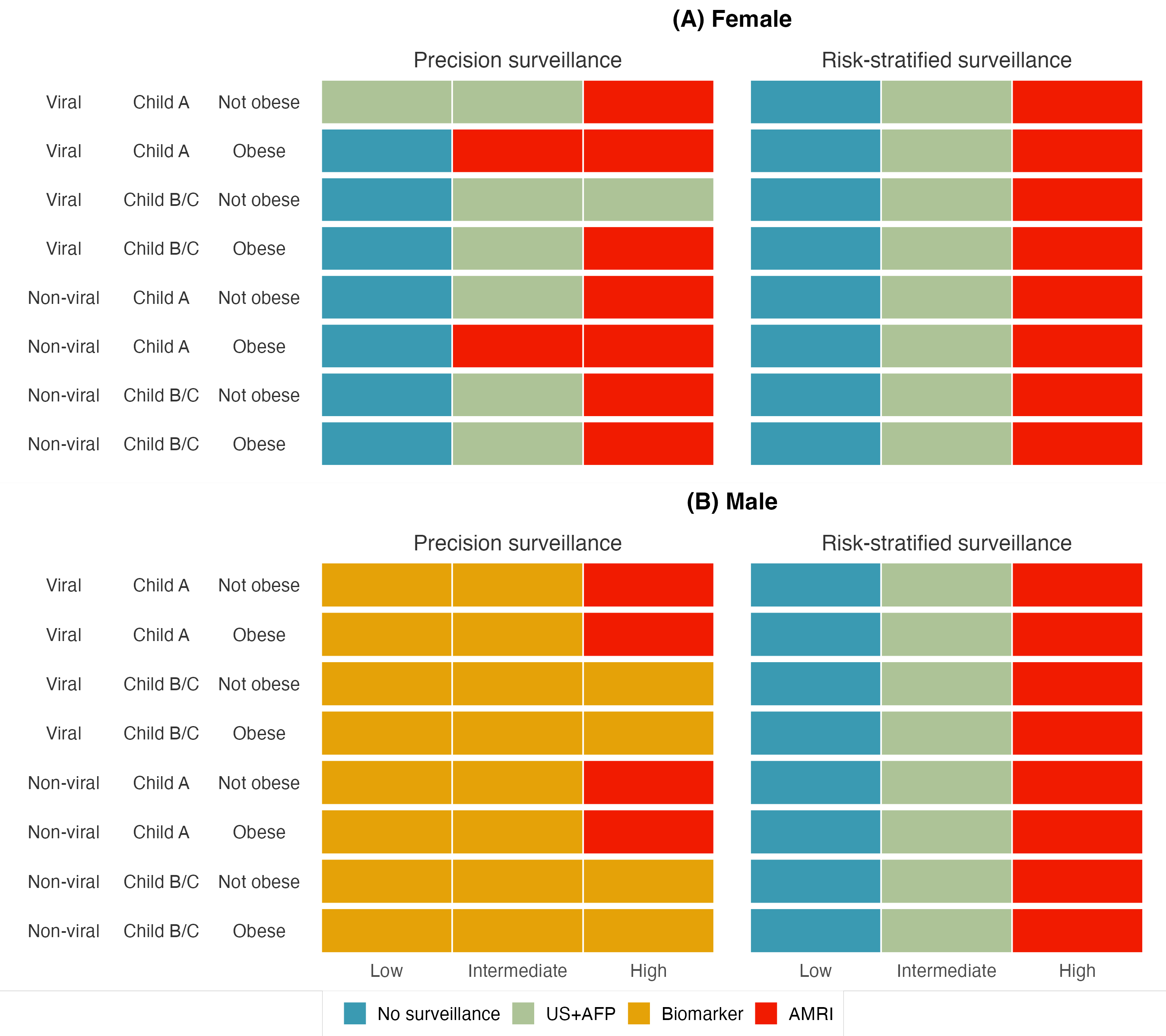


**Figure S5.** Number of HCC cases diagnosed per 100,000 population by stage and mode of detection.


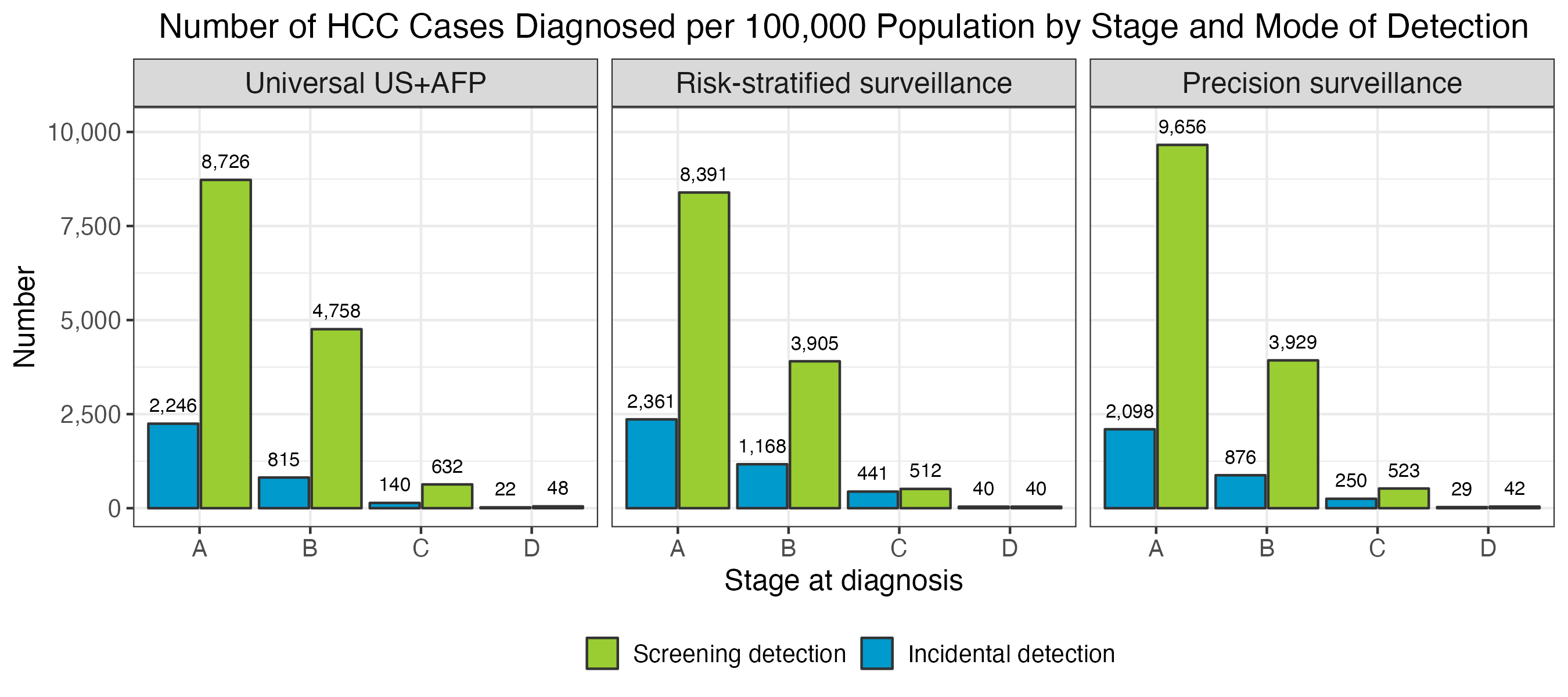


**Figure S6.** Difference in the number of HCC cases detected per 100,000 population of precision or risk-stratified surveillance compared with universal US+AFP.


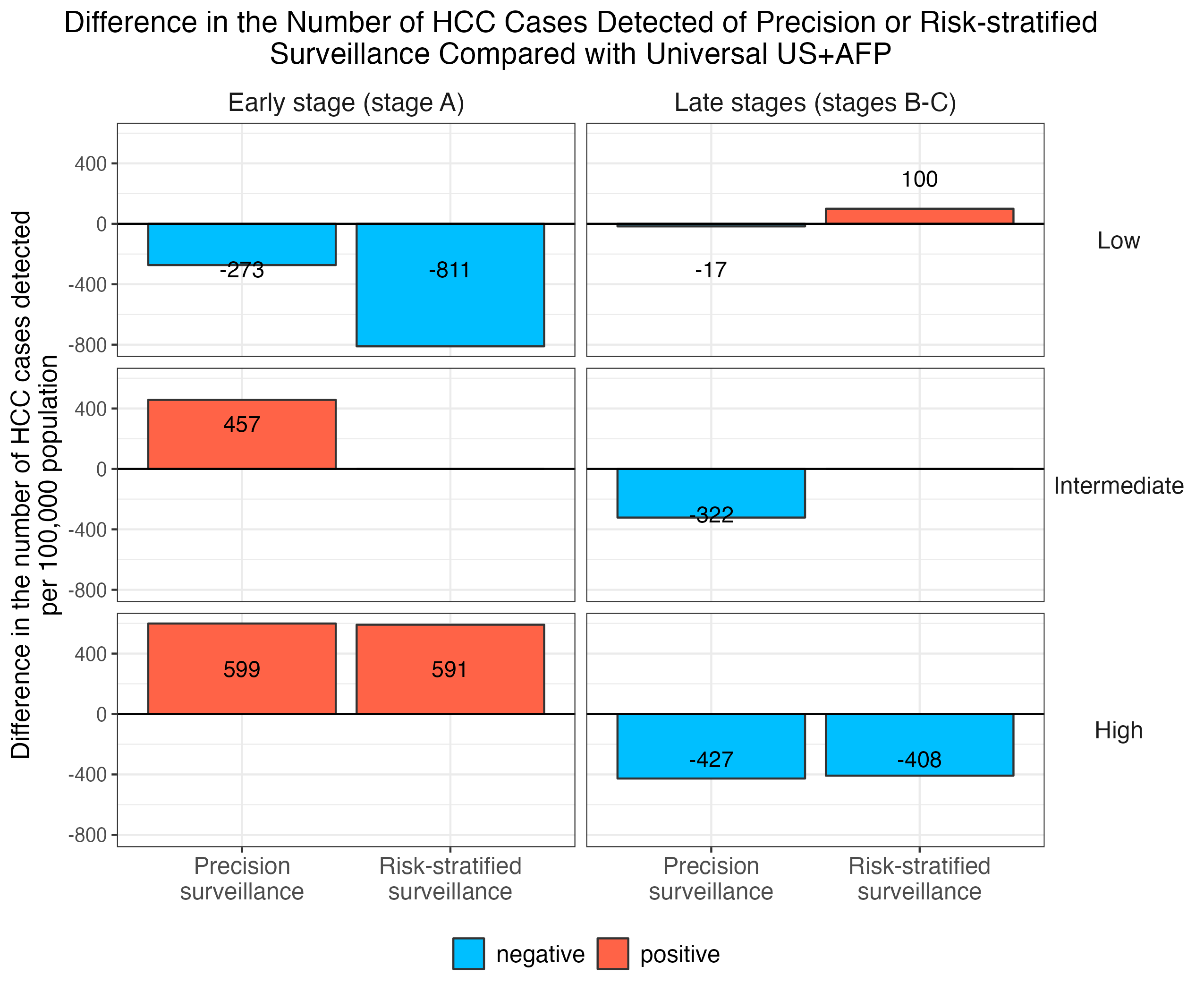


**Figure S7.** One-way sensitivity analysis among all test sensitivities and specificities, tornado diagrams compared between: (A) precision and universal US+AFP; (B) precision and risk-stratified surveillance; and (C) precision and no surveillance.


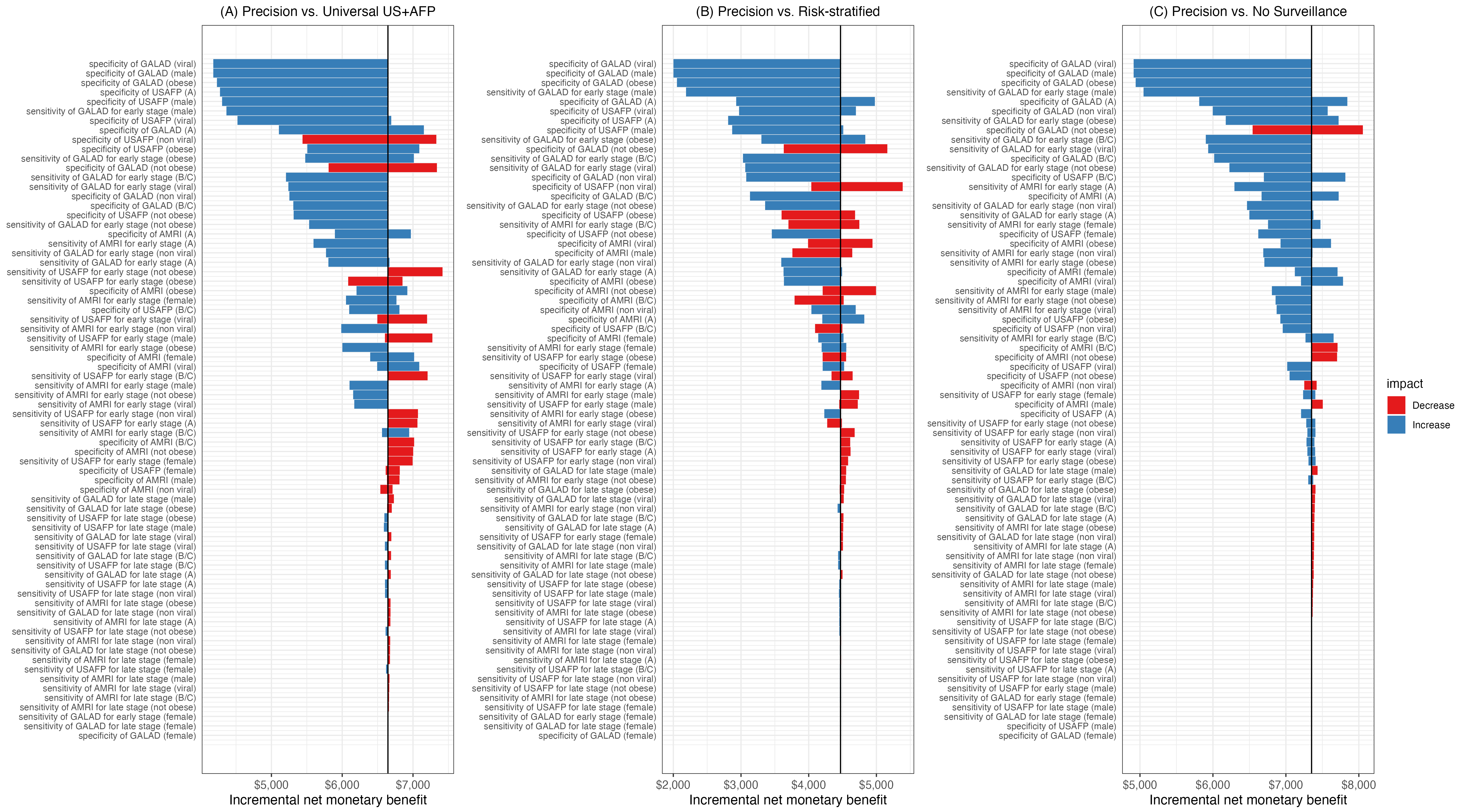


Positive incremental net monetary benefit (NMB) indicates a higher NMB for precision surveillance than the comparator.

**Figure S8.** Cost-effectiveness acceptability curve for head-to-head comparison between each surveillance strategy and no surveillance.


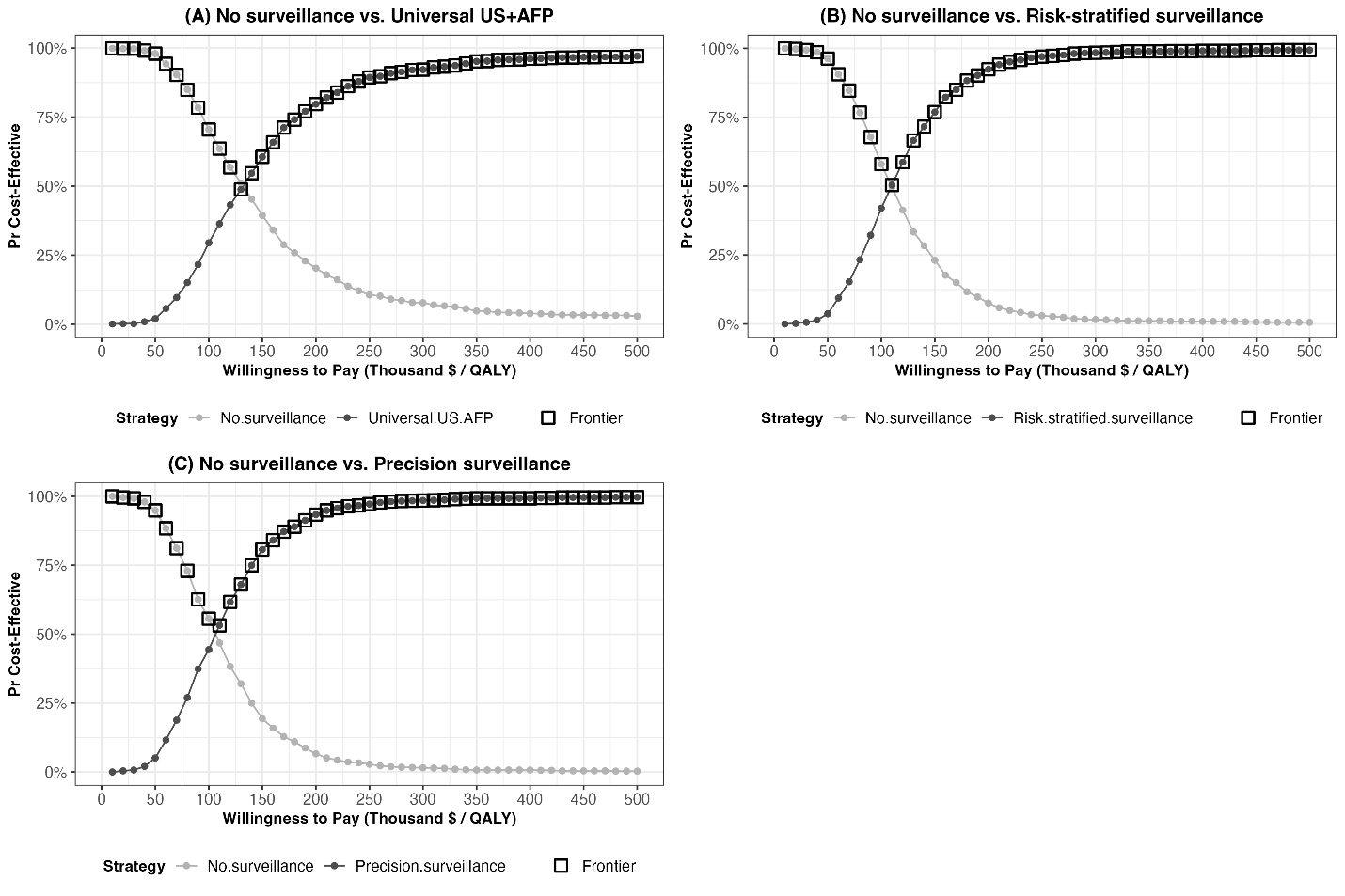


# **SUPPLEMENTARY REFERENCES**

1. Keeler E, Bell R. New DEALEs: other approximations of life expectancy. Medical Decision Making 1992;12:307-311.

2. Singal AG, Mittal S, Yerokun OA, et al. Hepatocellular Carcinoma Screening Associated with Early Tumor Detection and Improved Survival Among Patients with Cirrhosis in the US. Am J Med 2017;130:1099-1106 e1.

3. Wolf E, Rich NE, Marrero JA, et al. Utilization of hepatocellular carcinoma surveillance in patients with cirrhosis: A systematic review and meta-analysis. Hepatology 2020.

4. Daher D, El Dahan KS, Yekkaluri S, et al. Proportion Time Covered by Hepatocellular Carcinoma Surveillance in Patients with Cirrhosis. Official journal of the American College of Gastroenterology| ACG 2022:10.14309.

5. Shah C, Mramba LK, Bishnoi R, et al. Survival differences among patients with hepatocellular carcinoma based on the stage of disease and therapy received: pre and post sorafenib era. Journal of Gastrointestinal Oncology 2017;8:789.

6. Cheng A-L, Qin S, Ikeda M, et al. Updated efficacy and safety data from IMbrave150: Atezolizumab plus bevacizumab vs. sorafenib for unresectable hepatocellular carcinoma. Journal of hepatology 2022;76:862-873.

7. Brar G, Greten TF, Graubard BI, et al. Hepatocellular carcinoma survival by etiology: a SEER‐Medicare database analysis. Hepatology communications 2020;4:1541-1551.

8. Asrani SK, Hall L, Reddy V, et al. Comorbid chronic diseases and survival in compensated and decompensated cirrhosis: a population-based study. Official journal of the American College of Gastroenterology| ACG 2022;117:2009-2016.

9. Ioannou GN, Green P, Kerr KF, et al. Models estimating risk of hepatocellular carcinoma in patients with alcohol or NAFLD-related cirrhosis for risk stratification. J Hepatol 2019;71:523-533.

10. Ioannou GN, Green PK, Beste LA, et al. Development of models estimating the risk of hepatocellular carcinoma after antiviral treatment for hepatitis C. J Hepatol 2018;69:1088-1098.

11. Kanwal F, Khaderi S, Singal AG, et al. Risk Stratification Model for Hepatocellular Cancer in Patients With Cirrhosis. Clin Gastroenterol Hepatol 2023;21:3296-3304 e3.

12. Neumann PJ, Cohen JT, Weinstein MC. Updating cost-effectiveness—the curious resilience of the $50,000-per-QALY threshold. N Engl J Med 2014;371:796-797.

13. Low V, Macaulay R. Accounting for inflation within NICE cost-effectiveness thresholds. Expert Review of Pharmacoeconomics & Outcomes Research 2022;22:131-137.

14. Tzartzeva K, Obi J, Rich NE, et al. Surveillance imaging and alpha fetoprotein for early detection of hepatocellular carcinoma in patients with cirrhosis: a meta-analysis. Gastroenterology 2018;154:1706-1718. e1.

15. Yokoo T, Masaki N, Parikh ND, et al. Multicenter validation of abbreviated MRI for detecting early-stage hepatocellular carcinoma. Radiology 2023;307:e220917.

16. Singal AG, Tayob N, Mehta A, et al. GALAD demonstrates high sensitivity for HCC surveillance in a cohort of patients with cirrhosis. Hepatology 2022;75:541-549.

17. Schoenberger H, Chong N, Fetzer DT, et al. Dynamic changes in ultrasound quality for hepatocellular carcinoma screening in patients with cirrhosis. Clinical Gastroenterology and Hepatology 2022;20:1561-1569. e4.

18. Schad DJ, Vasishth S, Hohenstein S, et al. How to capitalize on a priori contrasts in linear (mixed) models: A tutorial. Journal of memory and language 2020;110:104038.

19. Simmons O, Fetzer DT, Yokoo T, et al. Predictors of adequate ultrasound quality for hepatocellular carcinoma surveillance in patients with cirrhosis. Alimentary pharmacology & therapeutics 2017;45:169-177.

20. Chong N, Schoenberger H, Yekkaluri S, et al. Association between ultrasound quality and test performance for HCC surveillance in patients with cirrhosis: a retrospective cohort study. Alimentary Pharmacology & Therapeutics 2022;55:683-690.

21. Marrero JA, Marsh TL, Parikh ND, et al. GALAD SCORE IMPROVES EARLY DETECTION OF HCC PRIOR TO THE DIAGNOSIS OF HCC: A PHASE 3 BIOMARKER VALIDATION STUDY, In HEPATOLOGY, WILEY 111 RIVER ST, HOBOKEN 07030-5774, NJ USA, 2021.
